# Personalized Revision Arthroplasty After Failed Medial Unicompartmental Knee Arthroplasty: A Contemporary Narrative Review

**DOI:** 10.3390/jcm15218577

**Published:** 2026-11-03

**Authors:** Paweł Skowronek, Adam Smolik, Filip Skowronek, Łukasz Dutka, Maria R. Skowronek, Klaudia Joniak, Michał J. Skowronek

**Affiliations:** 1 Orthopaedic and Trauma Department, Żeromski Specialist Hospital, Osiedle Na Skarpie 66, 31-913 Kraków, Poland; adamsmolik97@gmail.com (A.S.);; 2 Faculty of Medical Sciences in Katowice, Medical University of Silesia, Medyków 18, 40-752 Katowice, Poland; filipskowronek2002@gmail.com (F.S.);

**Keywords:** unicompartmental knee arthroplasty, revision knee arthroplasty, partial knee replacement, bicompartmental arthroplasty, patellofemoral arthroplasty, aseptic loosening, bone defect, clinical decision-making

## Abstract

Medial unicompartmental knee arthroplasty (UKA) is a bone- and ligament-preserving alternative to total knee arthroplasty (TKA) for selected patients with isolated medial compartment osteoarthritis, but it carries a consistently higher revision rate. The assumption that a failed UKA converts easily to a primary-standard TKA has not survived registry scrutiny, and conversion is now best regarded as a formal revision procedure. This contemporary narrative review, which is not a systematic review, evaluates revision strategies after failed medial UKA. Sources were identified through targeted literature searches and reference-list screening, and the strength of evidence was graded by level of evidence. Three pathways are considered: revision within the medial compartment, including bearing and single-component exchange; retention of a well-fixed, well-positioned medial UKA with addition of a patellofemoral arthroplasty or a lateral UKA; and conversion to TKA. Evidence quality differs sharply between pathways. Conversion to TKA is supported by registry and comparative cohort data, and medial UKA-to-UKA revision mainly by evidence of its high failure rate. Compartmental addition rests on one small dedicated series, one small multicentre comparative study, one descriptive multicentre series, one expert consensus and indirect data from primary bicompartmental arthroplasty. A four-step framework is proposed (failure mechanism, compartmental mapping, bone and ligament assessment, patient and centre factors); it is evidence-informed but not validated.

## 1. Introduction

Medial unicompartmental knee arthroplasty (UKA) is a bone- and ligament-preserving alternative to total knee arthroplasty (TKA) for appropriately selected patients with isolated medial compartment osteoarthritis. Compared with TKA, UKA has been associated with better functional patient-reported outcomes, a shorter hospital stay, and fewer complications, readmissions and deaths, whereas no consistent difference in pain has been demonstrated [1,2].

However, these advantages are accompanied by a consistently higher revision rate reported after UKA, as demonstrated across national joint registries. In a 27-year analysis of the Finnish Arthroplasty Register, Kaplan–Meier survivorship of UKA was reported as 89.4%, 80.6% and 69.6% at 5, 10 and 15 years, against 96.3%, 93.3% and 88.7% for cemented TKA over the same intervals [3]. A regional registry analysis of 6453 UKAs and 54,012 TKAs found a 15-year revision rate of 18.2% for UKA versus 6.2% for TKA, despite a lower perioperative complication rate (0.3% versus 0.6%) [4]. A systematic review of 56 studies reporting a minimum of ten years’ follow-up found that 8.8% of 30,140 UKAs had been revised, at a mean of 6.5 ± 2.6 years and a mean age at revision of 65.6 ± 5.6 years [5].

The response to those patients has been shaped by an assumption that has not held. Revision of a unicompartmental to a total knee arthroplasty was long presented as a straightforward procedure that would “buy time” while delivering results equivalent to a primary total knee replacement. A systematic review of the outcome literature concluded more than a decade ago that unicompartmental arthroplasty could not be regarded as a small procedure with primary-equivalent results when revised [6], and registry evidence accumulated since has not rehabilitated that view. Conversion also differs from primary TKA in several practical respects. Removal of the components and cement, and in particular tibial subsidence or collapse, can leave bone defects that require stems, augments or bone grafting [7,8,9]; restoration of the joint line and rotation may be difficult because the medial landmarks used by conventional instrumentation are disturbed [9]; and thicker polyethylene inserts are used more often than in primary TKA [8]. Nevertheless, most conversions can be performed with primary components: in a regional register audit of 201 failed UKAs, about one quarter required augmentation (26.4%) or bone grafting (25.9%), and only 8% required revision implants [9]. These demands are generally lower than those of revising a failed TKA [10], so that conversion lies between primary and revision TKA in surgical complexity (Section 6).

Two further findings have reshaped how that population should be approached. First, surgical volume is a modifiable determinant of failure. In the German Arthroplasty Registry (EPRD), 36,861 UKAs were analysed with a maximum follow-up of seven years; an annual volume of up to 25 UKAs carried a hazard ratio (HR) for aseptic revision of 1.86 (95% confidence interval (CI) 1.58 to 2.19) and a volume of 26 to 50 UKAs an HR of 1.43 (95% CI 1.22 to 1.69), both relative to 51 to 200 UKAs per year, while uncemented fixation carried an HR of 1.38 (95% CI 1.15 to 1.66) [11]. Nordic registry data have similarly linked low hospital volume to higher revision risk: among 14,496 cemented medial Oxford implants, hospitals performing 11 or fewer procedures per year had a 10-year implant survival of 80% (95% CI 78.0% to 82.0%), against 83% in higher-volume groups [12]. Second, a substantial proportion of UKA revisions may not be mechanically necessary at all. In a blinded radiographic review of 107 revised Oxford UKAs registered with the National Joint Registry, no reason for revision was visible in 67% of cases [13].

Against this background, the routine conversion of every failed UKA to a TKA deserves examination, given that conversion accounts for most revisions of failed UKA in registry data [4,14]. Conversion applies a tricompartmental solution to problems that are frequently unicompartmental or bicompartmental [15,16,17], and it permanently removes the bone stock and cruciate function that justified the index procedure. The counter-argument is equally serious: compartment-preserving strategies are supported by far weaker evidence, and repeat unicompartmental arthroplasty has been associated with re-revision rates several times higher than those after conversion [14,18,19]; applied outside narrow indications, such strategies risk reproducing the failure they were intended to correct. The choice of treatment after failed medial UKA should therefore be determined by the mechanism of failure, the condition of all three compartments, the extent of bone loss and the competence of the cruciate and collateral ligaments, rather than by the type of the index operation alone.

The purpose of this review is to define the contemporary failure modes of medial UKA, to describe the full spectrum of treatment options for the failed medial UKA, to appraise the quality and the limits of the evidence supporting each, and to propose an explicit, evidence-informed framework for choosing between them.

## 2. Literature Search and Evidence Selection

### 2.1. Type of Review

This is a contemporary narrative review based on targeted literature searches (Section 2.3). It is not a systematic review; it does not follow the Preferred Reporting Items for Systematic Reviews and Meta-Analyses (PRISMA) 2020 statement, and no claim is made that all eligible studies were identified or included. Accordingly, the evidence summaries in this review, including the statements on the strength of evidence at the end of Section 3, Section 4, Section 5 and Section 6, are qualitative appraisals by the authors. They are based on the highest level of directly relevant evidence, classified according to the levels of evidence for therapeutic studies [20], together with sample size and the directness of the evidence; no formal risk-of-bias assessment, meta-analysis or GRADE (Grading of Recommendations Assessment, Development and Evaluation) rating was performed. The manuscript was revised with reference to the six items of the Scale for the Assessment of Narrative Review Articles (SANRA) [21].

### 2.2. Designed Search Strategy (Not Executed)

To define the scope of the review explicitly, a structured search strategy was designed for PubMed/MEDLINE, Embase (Elsevier), Scopus and the Cochrane Library. It combines three concept blocks: (A) medial unicompartmental, unicondylar or partial knee arthroplasty; (B) failure, revision, conversion, reoperation, disease progression and named failure mechanisms; and (C) treatment destinations, including TKA conversion, repeat unicompartmental arthroplasty, bearing or component exchange, addition of patellofemoral arthroplasty, and addition of lateral unicompartmental arthroplasty. The strategy was not executed as a formal systematic search: no database-level record counts, deduplication or independent duplicate screening were performed, and no study-flow diagram is therefore presented. The complete database-specific Boolean strings are provided in Supplementary File S1 so that the search can be reproduced or updated in a future systematic review. The sources actually used in this review were identified as described in Section 2.3.

### 2.3. Source Identification and Selection

Sources were identified through targeted electronic database searches (PubMed/MEDLINE and Google Scholar), using combinations of the concept-block terms (for example, “unicompartmental knee arthroplasty”, “unicondylar” or “partial knee” combined with “revision”, “failure”, “conversion”, “bearing exchange”, “patellofemoral arthroplasty” or “bicompartmental”), supplemented by screening of the reference lists of relevant reviews and registry reports. Publications from database inception to 15 August 2026, the date of the last search, were eligible; sources identified during peer review were added up to October 2026. Eligibility criteria were: (i) population: failure, revision, conversion or compartmental addition after primary medial UKA; (ii) design: registry analyses, systematic reviews and meta-analyses, comparative cohorts, case series, consensus statements and, as indirect evidence only, comparative gait studies, with case reports excluded; (iii) no minimum follow-up, so that studies of early failure could be included; and (iv) peer-reviewed publications in English. Where several sources addressed the same question, priority was given to registry analyses, systematic reviews, comparative cohorts and larger series, and to more recent publications. Studies of mixed medial and lateral UKA populations were used only when results for medial UKA were reported separately or medial UKA formed the clear majority; revision of a primary lateral UKA was outside the scope of the review; and evidence from primary or simultaneous bicompartmental arthroplasty, or from TKA, was used only where direct evidence was lacking and is labelled as indirect throughout the text and tables. Sources were selected by A.S., F.S. and M.R.S. and checked by P.S., Ł.D., K.J. and M.J.S.; selection was not performed independently in duplicate, and disagreements were resolved by discussion and consensus, with the senior author (P.S.) deciding where consensus could not be reached. Methodological and definitional references (classifications, diagnostic definitions and reporting guidance) were cited where needed and were not subject to these criteria.

## 3. Failure Modes After Medial UKA

Accurate identification of the failure mechanism is the first determinant of treatment. The indication for revision should be based on a clearly defined cause of failure, such as component malposition, implant loosening, or progression of osteoarthritis in the lateral tibiofemoral or patellofemoral compartment [15,16]. Particular attention should be paid to identifying the source of pain, as revision surgery should not be undertaken in the absence of a clearly established diagnosis [13]. Unexplained pain alone should not be considered a sufficient indication for revision [13,22].

### 3.1. Overall Distribution

Evidence from two systematic reviews demonstrates a broadly consistent pattern in the mechanisms of UKA failure. Van der List et al., in an analysis of 3967 failures, identified aseptic loosening (36%) and progression of osteoarthritis in the unresurfaced compartments (20%) as the predominant failure modes. Unexplained pain (14%) and bearing dislocation (11%) were more frequently associated with mobile-bearing designs, whereas polyethylene wear (12%) and instability (12%) were more commonly observed in fixed-bearing designs [15]. Similarly, Tay et al., applying more restrictive inclusion criteria limited to studies comprising at least 250 patients and reporting at least 10 failures, identified osteoarthritis progression (30%) and aseptic loosening (24%) as the two leading causes of failure [16].

Registry data broadly agree while shifting the emphasis. In the EPRD (German Arthroplasty Registry), aseptic loosening accounted for 32.5% of revisions, with tibial component loosening alone accounting for 19.0%, followed by infection (11.0%) and progression of arthritis (10.0%) [11]. A single-centre analysis of 471 failed medial UKAs found other-compartment arthritis in 39.5% and aseptic loosening in 25.4%, at a mean of 6.1 years from the index procedure; 53.9% of failures occurred within five years and 22.9% beyond ten years [17].

Specialist revision practice shows a similar spread. In a multicentre series of 175 revisions of medial UKA performed across three institutions, the four commonest reasons for failure were femoral or tibial loosening (55%), progression of lateral or patellofemoral arthritis (34%), polyethylene failure (4%) and infection (3%), at a mean of 71.5 months from the index procedure (range 2 to 262) [23].

The variation between these figures is instructive rather than contradictory. Referral centres see more loosening [23]; population registries record a higher proportion of infection [11]; long-follow-up cohorts see more disease progression [16,17]. The distribution a surgeon encounters depends on where the patient presents, and no single set of percentages should be treated as canonical.

### 3.2. The Importance of Timing

Failure mode is strongly time-dependent, and this is clinically actionable. Tay and colleagues found that failures within six months were dominated by infection (40%), bearing dislocation (20%) and fracture (20%); failures between two and five years by osteoarthritis (OA) progression (33%), pain (21%) and aseptic loosening (17%); and failures beyond ten years overwhelmingly by OA progression (56%) [16].

The practical consequence is that early failures tend to be localised structural problems of the implant or its fixation, whereas late failures are more often biological progression in a compartment the index procedure never addressed, frequently with the original components still well fixed [16,17,24]. These two situations should not attract the same operation.

For treatment planning, four clinically distinct groups of failure should be separated. Early mechanical failure (bearing dislocation, early component loosening, malposition and tibial collapse) usually presents in the first postoperative years [7,16] and reflects a technical or implant-related problem; its treatment depends on whether the mechanism can be fully corrected locally (Section 4 and Section 7.3). Infection is governed by its acuity and by implant stability rather than by compartmental anatomy (Section 7.1). Periprosthetic fracture, typically an early complication [16,25], is managed according to fracture configuration and implant stability (Section 7.2). Late progression of osteoarthritis in the lateral tibiofemoral or patellofemoral compartment typically occurs with well-fixed medial components and is the only mechanism for which compartmental addition is a candidate strategy (Section 5). Because these groups require different revision strategies, each failure should be assigned to one of them before the operation is chosen.

### 3.3. Design- and Fixation-Specific Patterns

Bearing design shapes the failure profile. Rates of failure from wear were higher with fixed-bearing prostheses (5% versus 0.3%), whereas bearing dislocation occurred almost exclusively with mobile-bearing designs (14% versus 0%) [16]. Fixation matters similarly: aseptic loosening accounted for 27% of failures with cemented components and 4% with uncemented components in the same review [16], while the EPRD found uncemented fixation associated with an HR of 1.38 (95% CI 1.15 to 1.66) for aseptic revision [11]. These two observations are not contradictory (they describe different outcomes in different populations), but they should not be merged into a single claim.

Periprosthetic tibial fracture warrants specific comment. A systematic review and meta-analysis found the incidence of periprosthetic tibial fracture to be comparable between cementless and cemented UKA [25], whereas a single-centre analysis of 98 revisions associated uncemented fixation with revision within two years, owing to more periprosthetic fractures [26]. Fixation method is therefore not an established risk factor for this complication, but the evidence is not uniform.

### 3.4. Component Position

Malposition is a distinct and largely avoidable contributor. In 407 Oxford mobile-bearing medial UKAs followed for a mean of 91.7 months, the overall revision rate was 6.9%, most commonly for aseptic loosening (57.1% of revisions); malposition was the only factor associated with failure in a Cox proportional hazards model (*p* = 0.007; the hazard ratio was not reported), with five-year survival of 85.1% in malpositioned knees against 96.2% in correctly positioned knees (Kaplan–Meier comparison, *p* < 0.001), corresponding to five-year failure proportions of 14.9% and 3.8%, and most revisions occurring within three years [27].

### 3.5. Tibial Collapse

Medial tibial collapse deserves separate identification because it produces disproportionate bone loss. In a consecutive series of 32 UKA-to-TKA revisions, medial tibial collapse was the predominant failure mode in 15 knees (47%); 87% of these were all-polyethylene designs, 7 of 15 failed within 16 months, and reconstruction required stems, augments, screws and cement [7]. Increased posterior tibial slope was associated with posterior tibial collapse, although that series does not define a threshold value [7]. Thresholds for the posterior slope of the tibial component have been reported in other cohorts of medial UKA, although for overall survival rather than for collapse specifically: a slope greater than 5°, or a change in more than 2° from the native slope, was associated with reduced survival in 559 medial UKAs [28], and a slope greater than 7° has been advised against, particularly when the anterior cruciate ligament is deficient [29]. Because most collapses in that series occurred with all-polyethylene tibial components, the extent to which metal-backed designs reduce this risk cannot be quantified from the available data; when collapse occurs, it is associated with more complex reconstruction than other failure modes [7].

### 3.6. Unexplained Pain as an Indication for Revision

Pain without an identifiable structural cause is the most difficult indication in this field, and the evidence suggests it is over-treated. A National Joint Registry analysis of 402,714 primary knee arthroplasties (366,965 TKA and 35,749 UKA) found that unexplained pain accounted for 23% of UKA revisions versus 9% of TKA revisions; the five-year revision risk for unexplained pain was 1.6% after UKA and 0.2% after TKA (adjusted HR 6.76, 95% CI 5.84 to 7.83) [22]. Blinded radiographic review reinforces the point: in 107 revised Oxford UKAs, no radiographic reason for revision was identifiable in 67% [13], and in a further analysis of 98 revised UKAs by two independent reviewers, no radiographic reason for revision was identified in 34%, the indications for UKA had been satisfied in only 45%, and more than 40% of revisions were judged potentially avoidable [26].

The inference is not that these patients have no symptoms. It is that revising a well-fixed, well-positioned implant in the absence of a demonstrable structural target exposes the patient to a larger operation without addressing the source of pain. A structured diagnostic approach to these patients is outlined in Section 7.4.

Strength of evidence (qualitative) for Section 3: observational evidence of Level III and IV [20], including large registry analyses (Level III). Failure-mode percentages derive from study-level pooling across heterogeneous definitions and should be read as approximate.

Revision Strategies and Outcomes After Failed UKA.

Table 1 and Table 2 summarise the three treatment pathways and the principal studies informing this review.

## 4. Revision UKA to UKA (Within the Medial Compartment)

Revision confined to the medial compartment (bearing exchange, single-component revision or revision of both medial components) is conceptually the most conservative response to failure. The evidence indicates that its applicability is narrow.

### 4.1. Isolated Bearing or Insert Exchange

The only defensible indication is a failure mechanism that is entirely explained by the bearing itself and is fully correctable by replacing it: polyethylene wear in a metal-backed fixed-bearing implant with both metal components rigidly fixed and correctly positioned, or dislocation of a mobile bearing in which component position is confirmed satisfactory and the underlying soft-tissue imbalance can be corrected.

Durability is limited. Isolated insert exchange for polyethylene wear in metal-backed UKA has been reported, in a series of 20 knees, with a 12-year survival of 71.3% for revision for any reason and 93.3% for conversion to TKA [39]. In our view, wear rarely occurs in isolation: by the time it is symptomatic, occult loosening or progression in another compartment is often already present.

**Figure 1:**
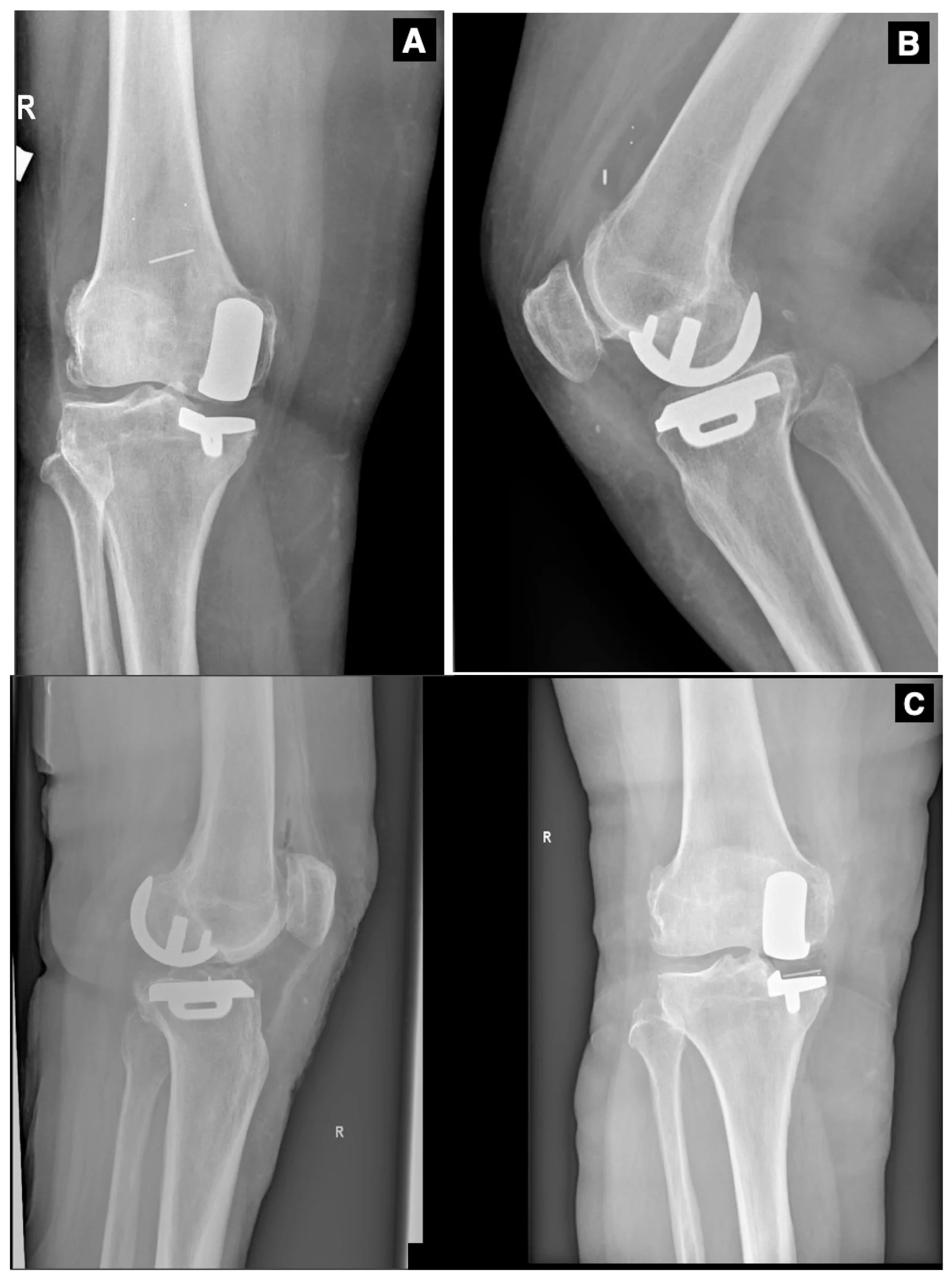
Mobile-bearing exchange for traumatic bearing dislocation in a well-fixed and correctly positioned medial UKA. (**A**) Preoperative anteroposterior and (**B**) lateral radiographs; (**C**) postoperative lateral and anteroposterior radiographs after bearing exchange. Patient: female, aged 62 years at revision, 2 years after the index UKA; follow-up 5 years.

Mobile-bearing dislocation is the more intuitive indication, but it is frequently a symptom rather than a diagnosis. Where dislocation is caused by component malposition or by flexion–extension gap mismatch, bearing exchange alone will not prevent recurrence, and this is reflected in outcome data: recurrent polyethylene dislocation accounted for 28% of re-revisions after repeat unicompartmental revision in the largest comparative series [18] (Figure 1).

### 4.2. Single-Component Revision

**Figure 2:**
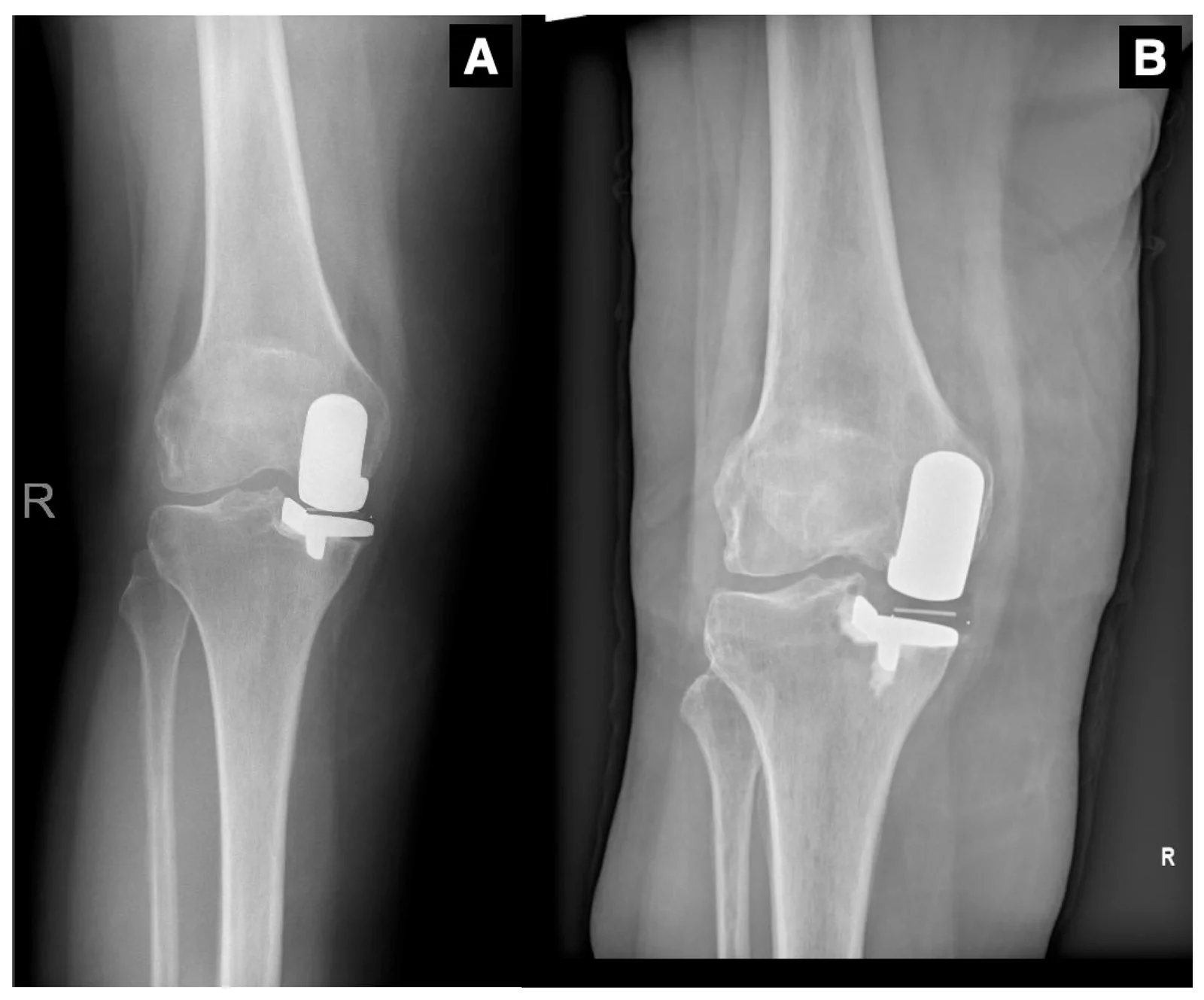
Isolated tibial component revision for aseptic loosening, with retention of a well-fixed and correctly positioned femoral component. The lateral tibiofemoral and patellofemoral compartments were preserved without significant osteoarthritic changes. (**A**) Preoperative and (**B**) postoperative anteroposterior radiographs. Patient: female, aged 67 years at revision, 4 years after the index UKA; follow-up 7 years.

Replacing one loose component while retaining a well-fixed counterpart has not been studied as a discrete category in the unicompartmental setting. The rationale rests on analogous total knee literature, where a systematic review of isolated femoral or tibial component revision reported clinical outcomes and re-revision rates broadly similar to those of full revision, except when revision was performed for wear or instability, although the quality of the included studies was low [40]. This is indirect evidence from a different construct and a different population, and the extrapolation must be stated rather than assumed. Since the tibial component is the more frequently loosened element in UKA [11], isolated tibial revision with femoral retention is the most plausible application, conditional on radiological confirmation that the femoral component is stable and correctly positioned (Figure 2). 

### 4.3. Revision of Both Medial Components

Registry evidence is consistent and unfavourable. In the Australian Orthopaedic Association National Joint Replacement Registry, 1948 revisions of primary UKA were analysed: when a UKA was revised to another UKA the three-year cumulative percent revision, excluding revisions for infection, was 28 (95% CI 22 to 36) for major and 30 (95% CI 22 to 40) for minor revisions, against 10 (95% CI 8.3 to 12) when the UKA was converted to TKA, the latter comparable to 12 (95% CI 9.0 to 15) for primary TKA revised at both components; the age- and sex-adjusted HR for re-revision was 2.6 (95% CI 1.9 to 3.6) for major UKA-to-UKA revision versus conversion, and 4.8 (95% CI 3.1 to 7.5) in the first 1.5 years for minor revision [14]. In the New Zealand Joint Registry, the revision rate after conversion of a failed UKA to a further UKA (31 knees) was reported as 13 times that of primary TKA, which had a rate of 0.48 revisions per 100 component-years; the source does not report the rate itself or a confidence interval, but the ratio corresponds to approximately 6.2 revisions per 100 component-years [19]. In a regional registry, 16 of 35 repeat unicompartmental revisions (45.71%) required re-revision, against 61 of 485 conversions to TKA (12.58%) [4], a crude risk ratio of 3.63 (95% CI 2.36 to 5.59) calculated for this review, which is not adjusted for follow-up time.

The most detailed comparative dataset comes from a single institution: among 241 knees revised between 1995 and 2022, two-year survivorship free of any re-revision was 96% after conversion to TKA and 75% after repeat unicompartmental revision, an absolute difference of 21 percentage points, with two-year survivorship free of any reoperation of 92% and 69%, respectively [18]; the source reports *p* < 0.0001 (Kaplan–Meier comparison) but no confidence intervals. In this series, 17 of the 24 repeat unicompartmental revisions (71%) were performed for acute PJI, so the comparison is confounded by indication. Five of seven patients requiring re-revision in the repeat-unicompartmental group were ultimately converted to TKA.

Against this stands one favourable series. In a multicentre French case–control study, 36 of 425 revised UKAs were managed with a further UKA; 3 (8.33%; 95% CI 1.75% to 22.47%, exact binomial) required iterative revision for aseptic loosening, at 8 months, 9 months and 3 years, and mean International Knee Society knee and function scores at a mean of 8.3 years were 93.81 ± 9.26 and 90.77 ± 15.41 [32]. The authors note that a meaningful Kaplan–Meier survival analysis was not possible in this cohort, and that the results reflect meticulous selection.

These findings are less contradictory than they appear. The registry and institutional series describe what happens when repeat unicompartmental revision is applied across a range of indications; the French series describes what happens when it is applied only where the failure is isolated, mechanically explicable, fully correctable, and unaccompanied by bone loss or ligament insufficiency.

Revision within the medial compartment should be reserved for a narrow group: an isolated, mechanically explicable and fully correctable failure, with the retained components confirmed well fixed and correctly positioned, competent collateral and cruciate ligaments, preserved bone stock, and no progression in the other compartments. Patients must be counselled explicitly that the risk of further revision is substantially higher than after conversion to TKA.

Strength of evidence (qualitative) for Section 4: consistent Level III registry and comparative evidence demonstrating a higher risk of re-revision; Level IV case-series evidence supporting favourable outcomes in carefully selected patients; and no direct evidence specifically evaluating isolated single-component revision in failed UKA.

## 5. The Compartmental Approach: Addition of a Second Partial Arthroplasty to a Well-Functioning Medial UKA

When failure results from progression of osteoarthritis in a previously unaffected compartment rather than from failure of the medial UKA itself, a compartment-preserving approach may be considered. Two strategies are available: addition of a patellofemoral arthroplasty (PFA) or addition of a lateral UKA. Although both aim to preserve a well-functioning medial UKA, they differ in their indications, surgical technique and supporting evidence and are therefore discussed separately. Neither strategy is currently supported as standard treatment. Direct evidence is limited to one small dedicated case series [31], one small multicentre comparative study of staged bicompartmental arthroplasty [24], a descriptive multicentre series [34] and an expert consensus statement [30].

### 5.1. Selection Criteria for Both Strategies

- The medial UKA is radiographically well fixed, correctly positioned in the coronal and sagittal planes, and clinically asymptomatic.
- Infection has been excluded.
- The anterior cruciate ligament is intact and functional, and the collateral ligaments are competent.
- Symptomatic osteoarthritis is limited to one unresurfaced compartment.
- The remaining third compartment is preserved.
- Alignment is acceptable or correctable without additional bony procedures.

### 5.2. Pathway B1: Addition of Patellofemoral Arthroplasty Following Medial UKA

Indication. Symptomatic progression of patellofemoral OA in the presence of a well-functioning medial UKA. A European Knee Society Delphi consensus identified six indications for adding PFA to a UKA: a well-functioning UKA with symptomatic patellofemoral OA progression, full-thickness lateral patellofemoral cartilage loss, intact ligaments including the ACL, preserved lateral tibiofemoral cartilage (≤ Ahlbäck grade 1), flexion ≥100° with ≤5° extension deficit, and older patients with increased comorbidities [30].

Two features of that statement deserve emphasis because they are counter-intuitive. The cartilage loss must involve the lateral facet, not simply “the patellofemoral joint”; and the consensus positions the procedure for older, comorbid patients, in whom the reduced magnitude of surgery is the principal advantage, not for young high-demand patients.

Technique and implant compatibility. The critical technical consideration is the interface between the retained medial femoral component and the new trochlear component. Impingement, a step-off at the junction, or overhang of the trochlear shield onto the medial condyle will produce anterior pain and can cause bearing or component damage. Preoperative planning should establish the identity and size of the in situ implant. Where the two components are of different systems, the geometry of the junction must be assessed directly at operation, and the surgeon must be prepared to abandon the strategy. Computer-navigated or robotic assistance may help in mapping the retained component and planning the trochlear resection, although no comparative evidence supports this in the addition setting specifically; in revision knee arthroplasty more broadly, technology assistance has been associated with fewer radiographic alignment outliers after revision TKA, with mixed results after revision UKA [38]. The recommendations in this paragraph are therefore based on technical principles and expert opinion rather than on outcome data; neither the EKS consensus [30] nor other sources identified for this review report outcomes related to implant compatibility in this setting.

Outcomes. We identified no direct outcome series in the sources available to this review evaluating patellofemoral arthroplasty added to a retained medial UKA. The available evidence is therefore indirect and derives primarily from studies of primary bicompartmental arthroplasty and primary PFA. For example, a non-designer series of 27 third-generation primary PFAs in 23 patients (92.6% women; mean age 51.7 years) reported five-year survival of 100% free of revision and 96.3% free of reoperation, with the OKS improving from 24.7 to 39.2 at a mean of 4.3 years [41]; these data cannot establish the efficacy or survivorship of staged PFA addition following medial UKA. In a matched cohort study comparing 25 primary modular bicompartmental arthroplasties with 50 primary TKAs, bicompartmental arthroplasty was associated with better WOMAC function (97.1 versus 89.7, *p* < 0.001), Knee Society function (90.0 versus 80.0, *p* = 0.003), range of motion (125.0° versus 120.0°, *p* = 0.004), Forgotten Joint Score-12 (89.6 versus 53.1, *p* < 0.001), and return to sport (71.6% versus 56.5%, *p* = 0.039), but with a longer operative time (105.0 versus 67.5 min, *p* < 0.001) [36]. Similarly, an instrumented gait study comparing 16 patients after primary medial bicompartmental arthroplasty with 20 primary TKA patients and 19 matched healthy controls demonstrated higher walking speed and more physiological gait parameters after bicompartmental arthroplasty [35]. These findings provide only indirect support and should not be extrapolated as evidence of clinical efficacy or implant survivorship for staged addition of PFA to a retained medial UKA.

**Figure 3:**
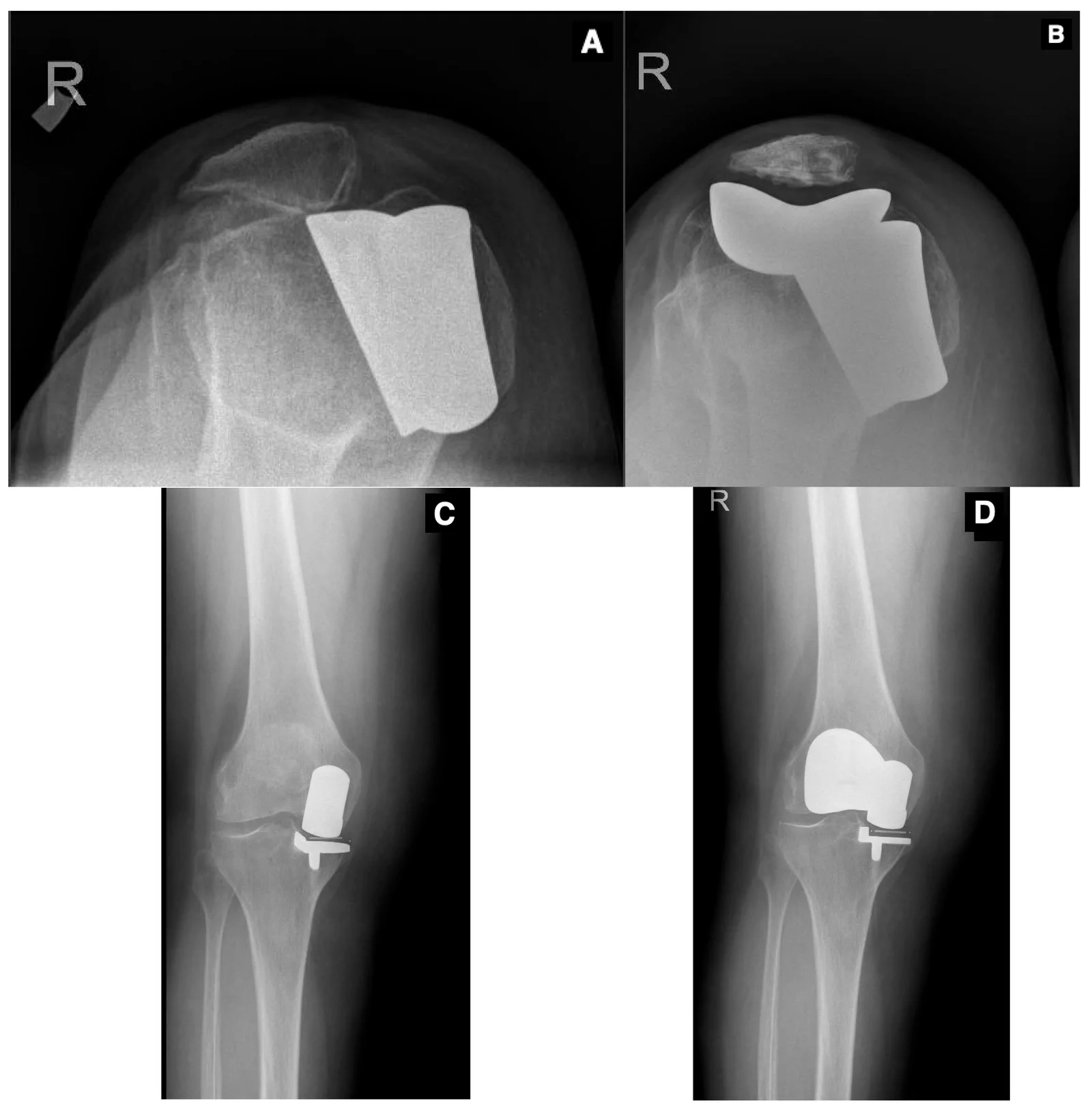
Addition of a patellofemoral arthroplasty for progression of patellofemoral osteoarthritis in the presence of a well-fixed and correctly positioned medial UKA, with preservation of the lateral tibiofemoral compartment without significant osteoarthritic changes. (**A**) Preoperative and (**B**) postoperative skyline views; (**C**) preoperative and (**D**) postoperative anteroposterior radiographs. Patient: female, aged 71 years at revision, 7 years after the index UKA; follow-up 6 years.

Complications and limitations. The specific risks are component impingement, anterior knee pain from an imperfect trochlear junction, and progression in the remaining lateral compartment, which converts a bicompartmental construct into an indication for TKA. The evidence base is expert consensus and indirect data; the strength of evidence is correspondingly low (Level V and indirect evidence) (Figure 3). 

### 5.3. Pathway B2: Addition of a Lateral UKA Following Medial UKA

Symptomatic osteoarthritis progression isolated to the lateral tibiofemoral compartment in a knee with a well-fixed, correctly positioned and clinically satisfactory medial UKA, an intact anterior cruciate ligament, competent collateral ligaments, an acceptable patellofemoral compartment and correctable alignment. Addition of a lateral UKA while retaining the medial implant results in a staged bi-unicompartmental arthroplasty. Direct clinical evidence for this strategy remains limited. Pandit et al. reported 27 knees in 25 patients undergoing staged bi-unicompartmental arthroplasty for symptomatic lateral osteoarthritis progression following medial UKA. The lateral UKA was performed a mean of 8.1 ± 4.6 years after the medial UKA. At a mean follow-up of 4.0 ± 1.9 years, functional scores improved significantly compared with the preoperative state (paired *t*-test, *p* = 0.003; effect sizes were not reported), with no radiographic failures, further operations or revisions (0 of 27; exact 95% CI upper bound for the failure proportion 12.77%). Median hospital stay was 3 days (range 2 to 9), no transfusions were required, and no significant procedure-related complications were reported [31]. Subsequent multicentre comparative evidence provides further support for the broader concept of staged compartmental addition. Haffar et al. compared 27 staged bicompartmental knee arthroplasties (sBiKA) with 30 conversions to revision TKA performed for progressive osteoarthritis after partial knee arthroplasty. Importantly, patients selected for sBiKA had an intact pre-existing partial knee arthroplasty without loosening or wear. At a mean follow-up of 5.7 ± 3.0 years for sBiKA and 3.2 ± 2.0 years for revision TKA, improvement in the Knee injury and Osteoarthritis Outcome Score for Joint Replacement (KOOS Jr.) was comparable between groups, whereas postoperative Knee Society Objective and Function Scores were higher after sBiKA (90.4 ± 10 vs. 72.1 ± 20 and 80.3 ± 18 vs. 67.1 ± 19, respectively). Only one patient in the sBiKA group required reoperation for persistent pain. The authors reported comparable short- to mid-term survivorship between sBiKA and revision TKA, while acknowledging that longer follow-up is required to establish durability [24]; with 27 and 30 patients, however, the study could detect only large standardised differences (d ≥ 0.76 at 80% power), so comparability should not be read as equivalence. Additional supporting evidence comes from a French multicentre series of 418 failed UKAs. Although most failures were converted to TKA, 11 patients (2.6%) underwent compartment-preserving addition of an arthroplasty in another compartment, including ten contralateral UKAs and one patellofemoral arthroplasty. These cases were not analysed as a separate outcome cohort and therefore provide evidence of clinical use rather than of efficacy or durability [34]. Taken together, these studies support the feasibility of staged compartmental addition in carefully selected patients with a well-functioning existing partial knee arthroplasty and isolated progression of osteoarthritis. However, the evidence remains limited. The dedicated medial-to-lateral UKA series is small, single-centre and without a comparator or formal survivorship analysis, while the multicentre comparative study evaluated staged bicompartmental arthroplasty more broadly rather than exclusively lateral UKA addition following medial UKA. The available evidence therefore supports feasibility and favourable short- to mid-term functional outcomes, but does not establish long-term durability or superiority over conversion to TKA.

**Figure 4:**
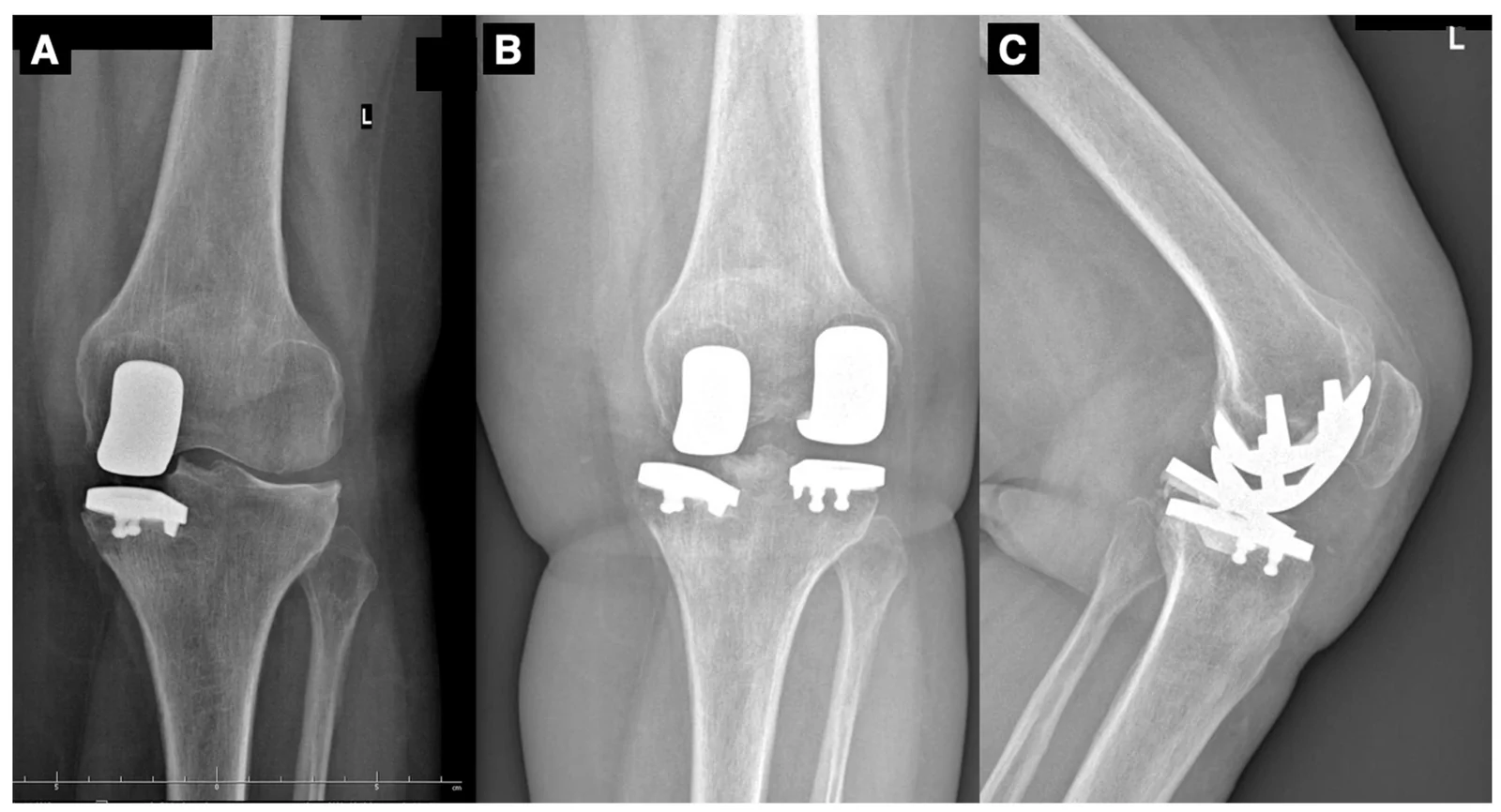
Addition of a lateral UKA for progression of lateral compartment OA with retention of a well-fixed and correctly positioned medial UKA and a preserved patellofemoral compartment without significant OA changes. (**A**) Preoperative anteroposterior radiograph; (**B**) postoperative anteroposterior and (**C**) lateral radiographs. Patient: male, aged 63 years at revision, 8 years after the index UKA; follow-up 3 years.

Both strategies are reasonable options in a narrowly defined group of patients, and both should be presented to patients as such: as procedures with a coherent rationale, encouraging but very limited outcome data, and no long-term evidence. Neither should be described as standard treatment (Figure 4).

Strength of evidence (qualitative) for Section 5: Level V expert opinion and indirect evidence for Pathway B1; for Pathway B2, one Level IV case series [31], one Level III comparative study of staged bicompartmental arthroplasty more broadly [24] and a descriptive multicentre series [34], supplemented by indirect evidence. No randomised or prospective comparative evidence exists for either.

## 6. Conversion of Medial UKA to Total Knee Arthroplasty

Conversion is the most frequently performed and best-evidenced response to a failed UKA. In the Australian registry, 1743 of 1948 revisions of primary UKA (89.48%) were conversions to TKA [14]; in a regional registry, 485 of 620 (78.23%) [4].

### 6.1. Indications

Conversion to TKA is indicated in the presence of multicompartmental osteoarthritis, significant bone loss, component loosening, malposition or subsidence, ACL or collateral ligament insufficiency, fixed or uncorrectable deformity, periprosthetic fracture requiring reconstruction, failure of previous compartment-preserving revision, or chronic PJI requiring staged revision. Conversion to TKA should also be the preferred option when the criteria for a compartment-preserving procedure are not met.

### 6.2. Is Conversion Equivalent to a Primary TKA?

It is not. In the New Zealand Joint Registry, the revision rate after conversion was four times that of primary TKA (1.97 versus 0.48 per 100 component-years, *p* < 0.05), and the mean Oxford Knee Score was significantly worse (30.02 versus 37.16, *p* < 0.01) [19]. A meta-analysis of five studies and 536 patients found primary TKA superior to converted UKA in WOMAC, Knee Society Score and range of motion (all *p* < 0.05), with conversion requiring more augments, stems, bone grafts and thicker polyethylene [8]. That analysis found no significant difference in revision rate, hospital stay or complications; this finding must not be read as equivalence, since five small studies provide little power to detect such differences.

The counterpoint comes from specialist practice. In a high-volume centre reporting 439 conversions, ten-year aseptic survivorship was 95.6% (95% CI 91.7 to 97.7), similar to that of primary TKA (95.8%, 95% CI 95.3 to 96.3) and higher than that of revision TKA (84.2%, 95% CI 80.5 to 87.3), with the aseptic failure mode of the original UKA having no effect on outcome (*p* = 0.80) [33]. Conversion outcomes therefore appear to sit between primary and revision TKA, with the position within that range determined substantially by the reconstruction achieved and by the experience of the operating team.

Re-revision after conversion is uncommon in specialist series: 9 of 175 knees (5.14%; 95% CI 2.38% to 9.54%; reported as 4.5% in the source) required further revision, at a mean of 48 months, during a mean follow-up of 75 months after conversion in a three-centre cohort, a rate of 1.23 revisions per 100 observed component-years [23].

It is also worth recording that revision of a UKA is generally less demanding than revision of a TKA. In a community registry comparing 68 revisions of UKA with 112 revisions of TKA, re-revision rates were similar (5.9% versus 6.3%), but revision TKA was consistently more complex by operative time, augmentation and stem use, and liner thickness; 39 of 68 revisions of UKA (57%) required no augmentation at all and were performed as primary TKAs [10].

### 6.3. Bone Loss and Reconstruction

The revision complexity of UKA-to-TKA conversion varies according to the mode of failure and the extent of bone loss. In an audit of 201 conversions from a regional arthroplasty register, augmentation was required in 50 cases with available reconstructive data, most commonly using tibial stems (35 cases), tibial wedges (24) and femoral stems (17). Bone grafting was required in 49 cases, predominantly in patients aged 60 years or older. A primary cruciate-retaining implant was used in 78% of cases with available implant data, while the remainder required cruciate-sacrificing or revision components [9]. The need for additional reconstruction is concentrated in failure modes associated with substantial bone loss. Medial tibial collapse may require stems, augments, screws and cement [7], with all-polyethylene tibial designs over-represented among these cases.

### 6.4. Balancing, Joint Line and Constraint

The following technical recommendations are based on general principles of revision knee arthroplasty and on published descriptions of conversion, rather than on comparative evidence [9,10,42]. Removal of the unicondylar implant alters the medial joint line and removes the landmarks on which conventional instrumentation depends. Restoration of the joint line should be referenced from the lateral femoral condyle and the fibular head rather than from the disturbed medial side. Flexion and extension gaps should be assessed after complete cement removal, since retained cement is a common cause of apparent tightness.

**Figure 5:**
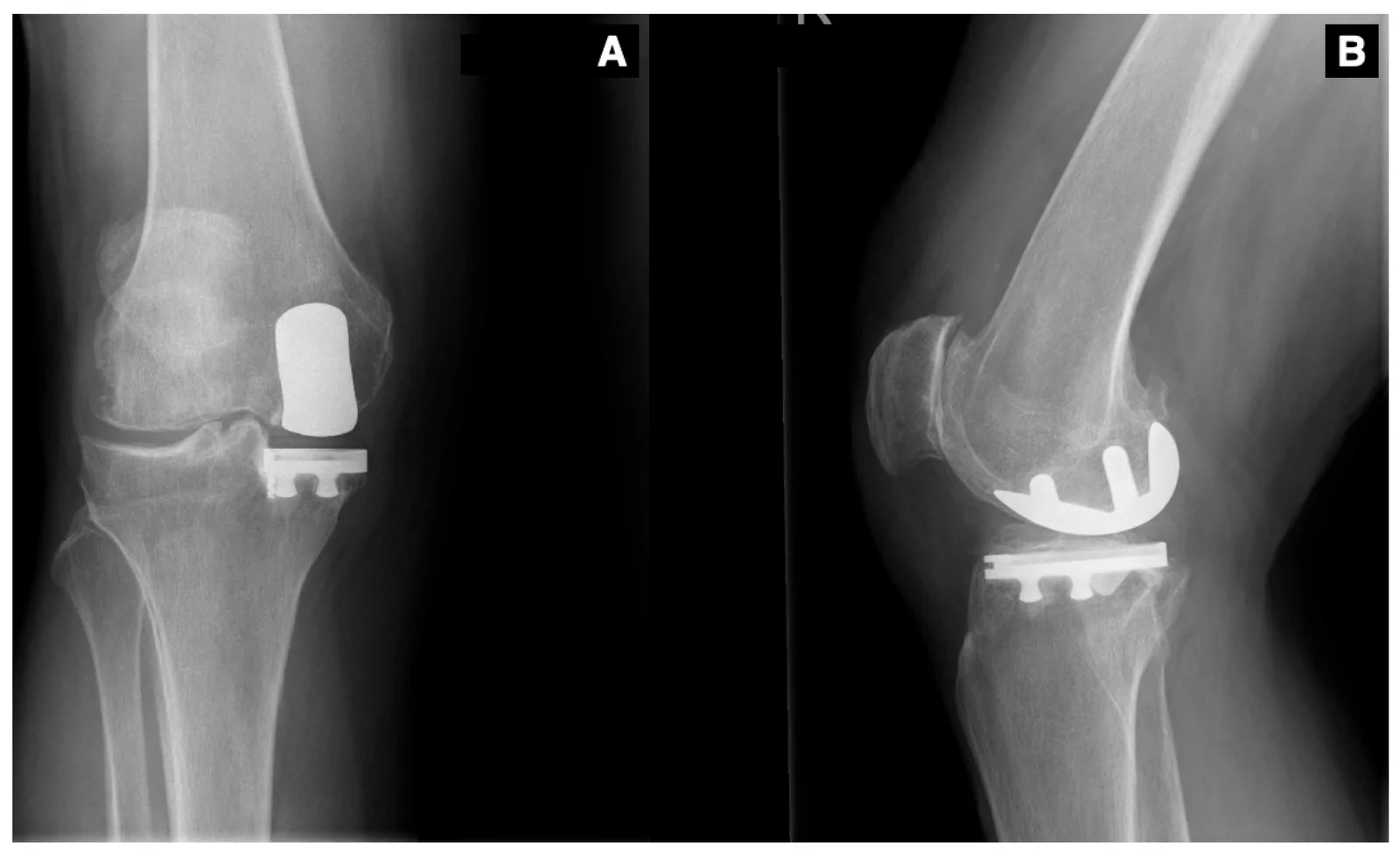

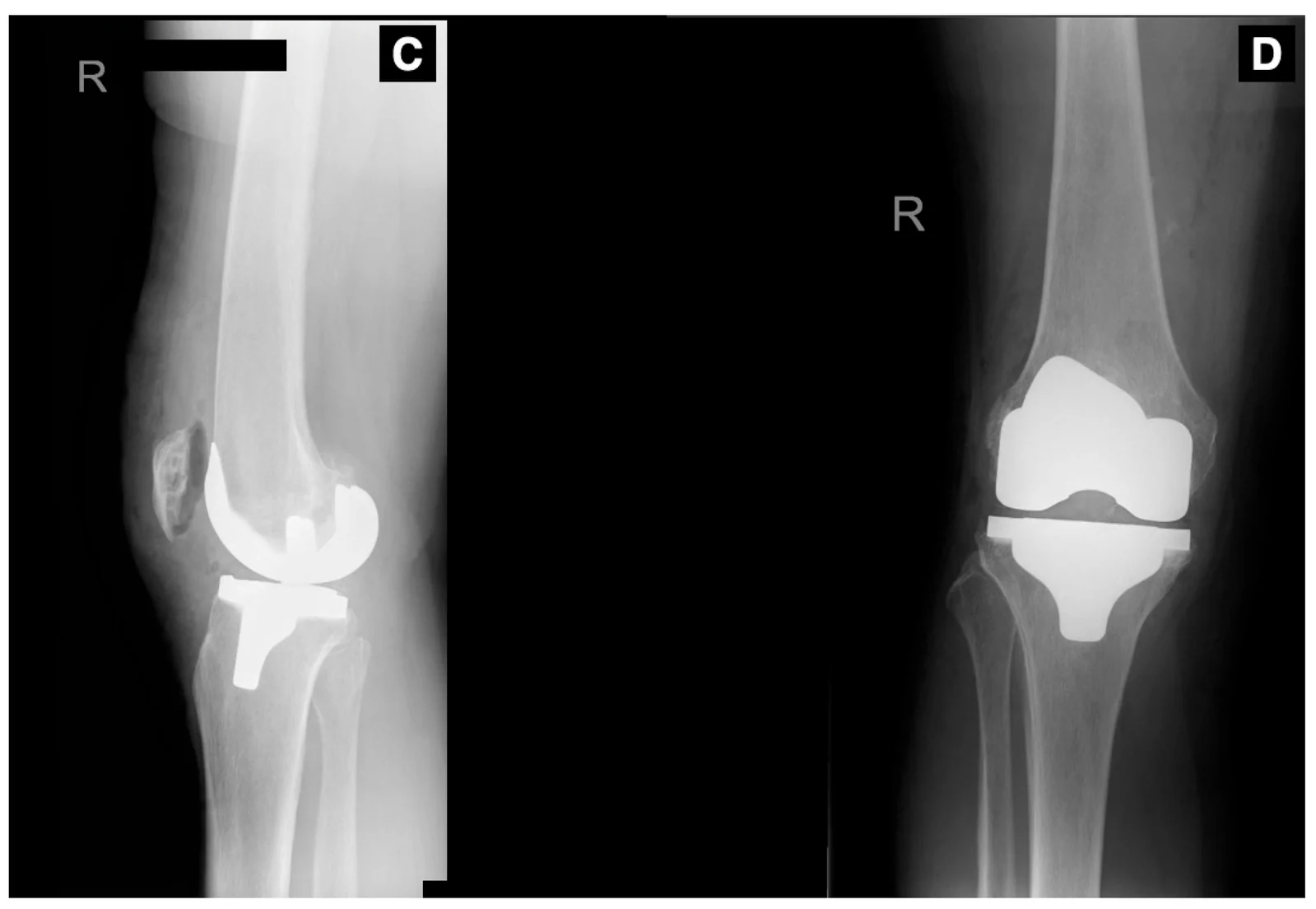
Conversion of a failed medial UKA to TKA for progression of osteoarthritis in the remaining compartments. (**A**) Preoperative anteroposterior and (**B**) lateral radiographs; (**C**) postoperative lateral and (**D**) anteroposterior radiographs. Patient: male, aged 73 years at revision, 11 years after the index UKA; follow-up 6 years.

**Figure 6:**
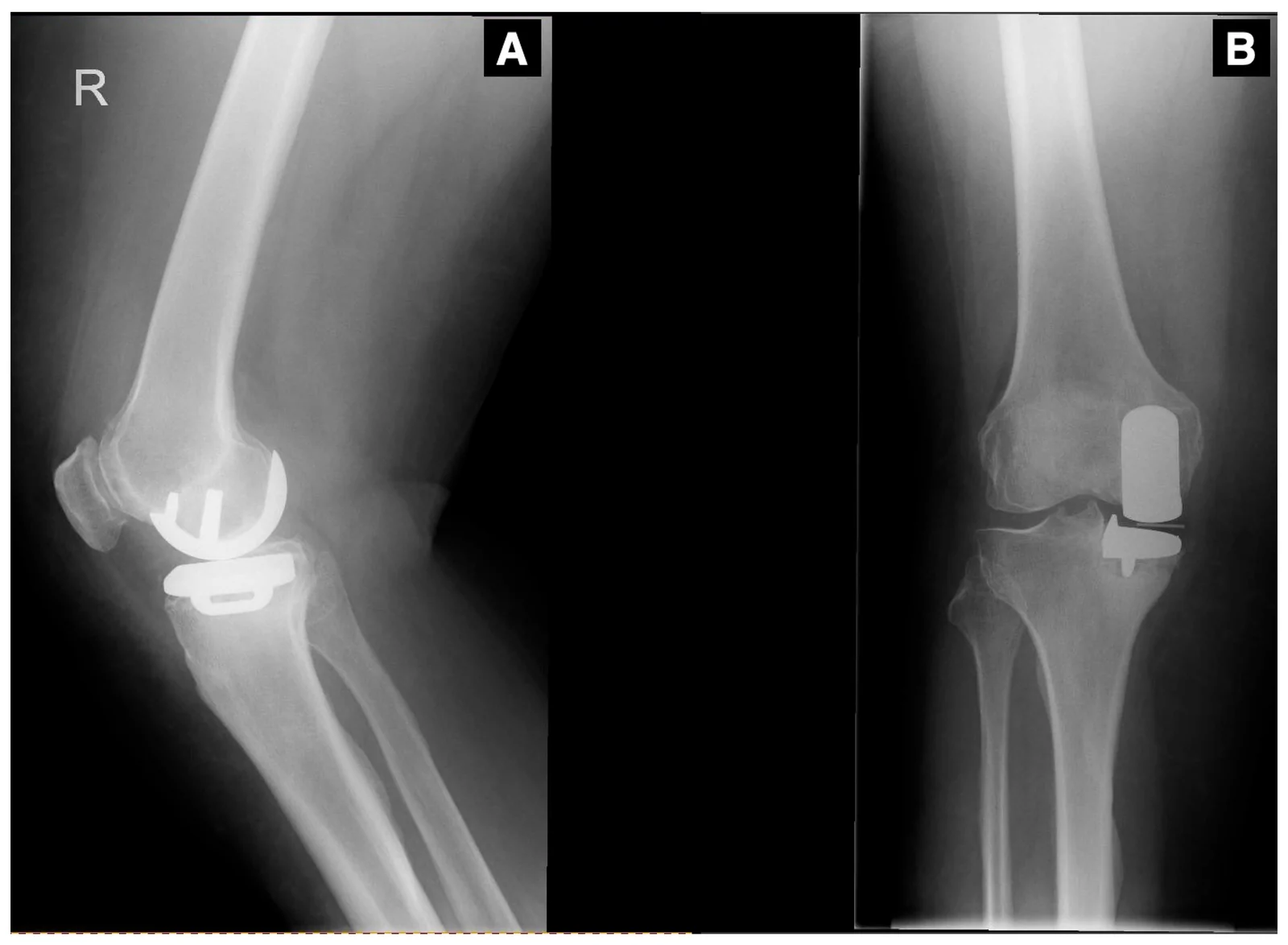

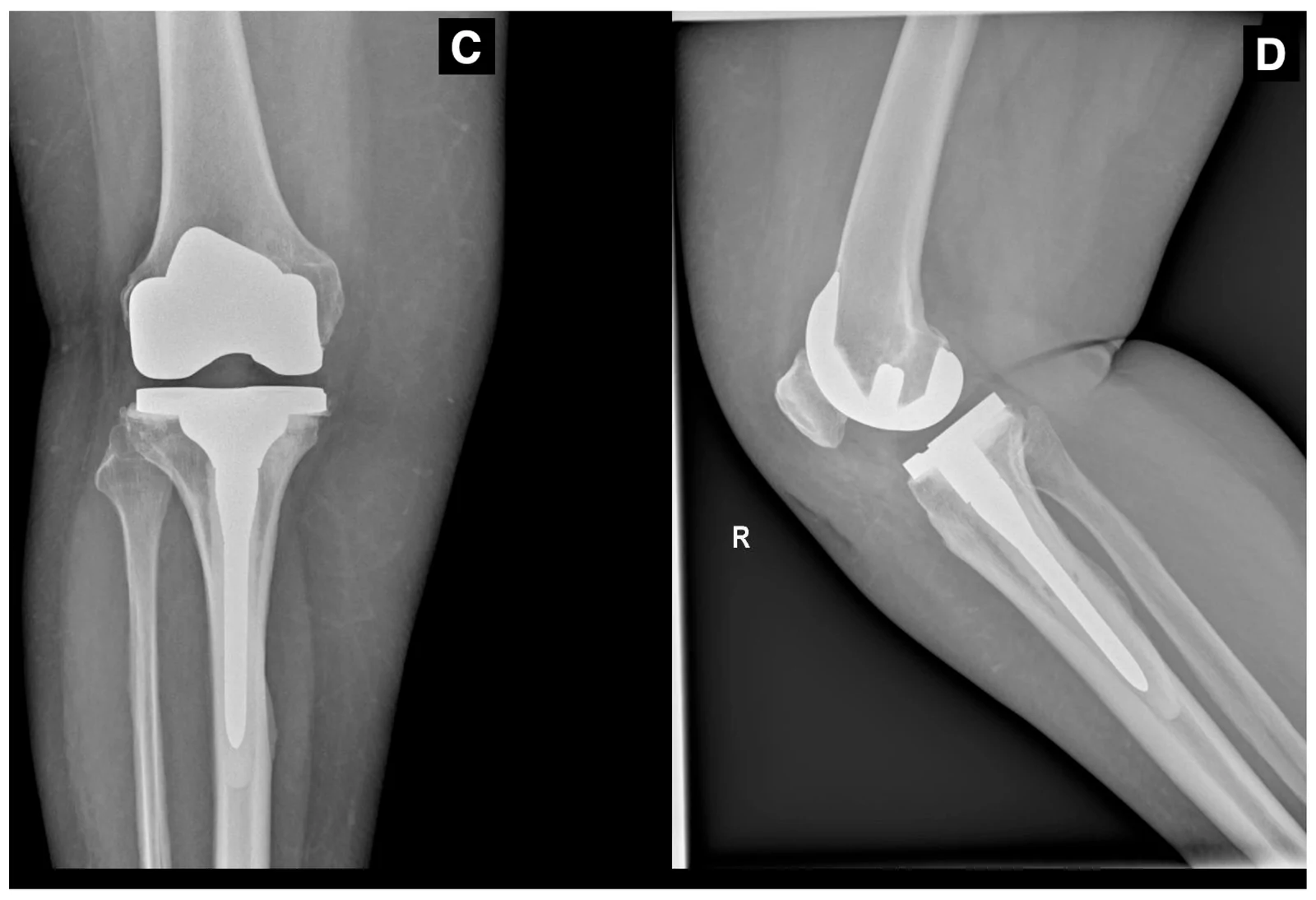
Conversion of a failed medial UKA to TKA for tibial component loosening with associated bone loss, requiring a tibial stem to achieve adequate implant fixation and stability. (**A**) Preoperative lateral and (**B**) anteroposterior radiographs; (**C**) postoperative anteroposterior and (**D**) lateral radiographs. Patient: female, aged 73 years at revision, 5 years after the index UKA; follow-up 7 years.

Constraint should follow the principle of using the least necessary. A cruciate-retaining implant is appropriate where the posterior cruciate ligament is competent and the gaps balance, and remains usable in the majority of conversions [9]. Posterior-stabilised designs are indicated where the posterior cruciate is deficient or the gaps cannot otherwise be balanced. Condylar-constrained implants are reserved for collateral insufficiency or for defects that leave the construct dependent on augmentation for stability. Metaphyseal cones or sleeves are required where AORI Type 2B or Type 3 defects leave insufficient bone for reliable fixation [43,44] (Figure 5 and Figure 6). 

Strength of evidence (qualitative) for Section 6: Level III registry and comparative cohort evidence, and one meta-analysis of comparative studies [8], for survivorship and function relative to primary TKA.

## 7. Special Failure Scenarios

Four situations are not adequately handled by a simple choice between the three pathways, and require their own logic.

### 7.1. Periprosthetic Joint Infection

Periprosthetic joint infection (PJI) after UKA is uncommon but represents a challenging cause of failure. In 36,861 UKAs in the German Arthroplasty Registry, the septic revision rate was 0.9% at seven years, and infection accounted for 11.0% of all revisions [11]. Risk factors for septic revision were grade III obesity (HR 1.83, 95% CI 1.04 to 3.24), male sex (HR 1.69, 95% CI 1.26 to 2.26) and an Elixhauser comorbidity score of 5 or more (HR 1.67, 95% CI 1.06 to 2.65) [11]. Sex had opposite associations with the two main failure modes in this registry: male sex was associated with a lower risk of aseptic revision (HR 0.75, 95% CI 0.66 to 0.84) [11]; sex-disaggregated outcomes of revision procedures after failed UKA are rarely reported and should be reported in future studies [45]. The diagnosis should be based on an internationally accepted definition, such as that of the European Bone and Joint Infection Society (EBJIS) [46].

Infection is governed by acuity, organism and soft-tissue status rather than by compartmental anatomy. Acute infection (early postoperative or acute haematogenous) with a susceptible organism, an intact soft-tissue envelope and well-fixed components may be treated by debridement, antibiotics and implant retention (DAIR), including thorough synovectomy and exchange of the modular bearing. In a multicentre series of 52 UKAs, DAIR controlled infection at one year in 80.8% of patients, success was not correlated with the time from index surgery to diagnosis, and synovectomy was associated with eradication [47]; a meta-analysis of nine studies (165 patients with medial or lateral UKA) reported a pooled infection-free survival after DAIR of 72% (95% CI 60% to 82%) and subsequent conversion to TKA in 34% (95% CI 17% to 57%) [48]. Chronic infection generally requires complete implant removal, radical debridement and staged conversion to TKA, frequently with an antibiotic-loaded articulating spacer; in 16 delayed or late UKA infections treated with a two-stage protocol, infection-free implant survival was 100% at a mean follow-up of 33.5 months, with one positive intraoperative culture managed by suppressive antibiotic therapy; constrained condylar implants were used in 10 of the 16 knees [49]. Implant removal and debridement may leave substantial tibial or femoral defects requiring stems, metallic augments or other reconstruction techniques [42].

The evidence urges caution about component-retaining strategies here. Among patients in whom repeat unicompartmental revision was performed for acute PJI, two-year survivorship free of re-revision was 67%, and infection was the commonest cause of re-revision after repeat unicompartmental revision overall (57% of re-revisions) [18]. Infection also dominates the earliest failures, accounting for 40% of revisions within six months [16].

### 7.2. Periprosthetic Fracture

Periprosthetic tibial fracture is uncommon and, importantly, occurs at a comparable incidence with cementless and cemented fixation (pooled 1.24%, 95% CI 0.64% to 2.41%, and 1.58%, 95% CI 1.06% to 2.36%, respectively); most fractures occur intraoperatively or within three months of surgery [25]. Management depends on implant stability and fracture configuration. A stable implant with a displaced fracture amenable to fixation may be treated by open reduction and internal fixation with the arthroplasty retained. A fracture involving or destabilising the tibial component requires conversion, usually with a stemmed tibial implant bypassing the fracture and augmentation of the resulting defect. Fracture is not an indication for a compartment-preserving strategy. These management principles are derived from general periprosthetic fracture practice and expert opinion; we identified no comparative evidence specific to fractures after UKA.

### 7.3. Mobile-Bearing Dislocation

Dislocation is the archetypal early mobile-bearing failure [16]. The decisive question is why the bearing dislocated. Where component position is confirmed satisfactory, and the cause is a correctable soft-tissue imbalance, bearing exchange (with upsizing if appropriate) is reasonable. Where malposition or gap mismatch is responsible, bearing exchange alone is likely to fail, and conversion to a fixed-bearing unicompartmental implant or to TKA should be considered, although no comparative data guide this choice. Recurrent dislocation accounted for 28% of re-revisions after repeat unicompartmental revision [18], which is the clearest available warning against treating the symptom rather than the cause.

### 7.4. Diagnostic Work-Up of Unexplained Pain

Pain without a structural target should not be revised. The diagnostic obligation is to exclude infection, referred pain from the hip or spine, nerve entrapment, complex regional pain syndrome, and central sensitisation, and to review whether the original indication for partial arthroplasty was sound. In practice, the work-up should address five questions. First, infection must be excluded: serum C-reactive protein (CRP) and erythrocyte sedimentation rate (ESR) should be measured and, if either is raised or clinical suspicion persists, the knee should be aspirated for synovial white cell count (WCC), polymorphonuclear (PMN) percentage and culture, interpreted against a current definition of PJI [46]. Second, fixation should be assessed on serial radiographs aligned to the tibial component under fluoroscopic control, because pre-revision radiographs were malaligned and difficult to interpret in 53% of revised Oxford UKAs in a registry sample [13] and a narrow, well-defined radiolucent line beneath the tibial component is a common physiological finding after Oxford UKA that is unrelated to pain and should not be interpreted as loosening [50]; computed tomography (CT) defines component position, subsidence and bone loss, and single-photon emission computed tomography combined with CT (SPECT/CT) may help to localise the source of symptoms when conventional imaging is inconclusive [51]. Third, limb alignment and the state of the lateral and patellofemoral compartments should be assessed on full-length standing and skyline radiographs. Fourth, ligament stability, including the anterior cruciate ligament, should be examined clinically and, where doubt remains, with stress radiographs. Fifth, extra-articular sources should be excluded, as listed above, and modifiable psychological factors, in particular kinesiophobia (fear of movement), should be assessed, for example with the Tampa Scale for Kinesiophobia; structured rehabilitation and exercise programmes (mean reduction in the Tampa Scale score of 7.95 points) and cognitive-behavioural interventions (6.12 points) reduced kinesiophobia after total knee arthroplasty in a meta-analysis of 16 randomised trials, with moderate- and low-certainty evidence, respectively, as rated by its authors [52], and an integrative model has been described [53]. When no structural cause is identified, non-operative management (physiotherapy with quadriceps strengthening, analgesia, activity modification, management of kinesiophobia and periodic re-evaluation) is the default, and revision should be reconsidered only if a structural cause becomes demonstrable, because revision for unexplained pain tends not to relieve the pain [13]. The registry and radiographic evidence set out in 3.6 indicates both that this indication is applied more readily to UKA than to TKA [22] and that most such revisions have no radiographic justification [13,26]. Where revision proceeds despite the absence of a structural target, the patient should be counselled explicitly that outcomes are likely to be poor irrespective of the strategy chosen [13].

## 8. A Proposed Framework for Personalized Decision-Making

The framework below is proposed and evidence-informed. It has not been prospectively validated, and it is not offered as a substitute for intraoperative judgement. It is intended to structure clinical reasoning and should not be regarded as a proven or validated clinical decision tool.

Before a decision is made, six items should be documented explicitly: the failure mechanism; the infection status; the fixation and position of each existing component; the location and severity of bone loss; the competence of the collateral ligaments, the anterior cruciate ligament and the extensor mechanism; and the state of both unresurfaced compartments. Where any of these cannot be established preoperatively, the operative plan must include the capacity to proceed to the more extensive option.

### 8.1. Step 1: Establish and Classify the Failure Mechanism

Failure falls into four mechanistic categories, each implying a different direction of treatment; unexplained pain is deliberately not one of them (Section 7.4):

- Partial mechanical failure: isolated polyethylene wear with both metal components well fixed and correctly positioned; isolated bearing dislocation without malposition; single-component loosening with the counterpart confirmed stable → consider the most limited intervention that fully corrects the mechanism.
- Complete mechanical failure of the implant: loosening of both components, tibial subsidence with bone loss, periprosthetic fracture involving the implant, or malalignment not correctable by exchange → full revision required.
- OA progression in an unresurfaced compartment, with the medial UKA radiologically sound and clinically satisfactory → consider compartmental addition.
- Infection: acuity-governed protocol, independent of compartmental anatomy.

### 8.2. Step 2: Map the Compartments

Define the status of all three compartments (medial, lateral tibiofemoral and patellofemoral) using a stepwise imaging protocol. Standing weight-bearing anteroposterior and lateral radiographs, a skyline view of the patellofemoral joint and a full-length standing radiograph of the limb form the baseline. Valgus and varus stress radiographs show whether the lateral joint space is preserved, whether the deformity is correctable and whether the collateral ligaments are competent, although they reflect cartilage thickness rather than cartilage quality [54]. CT, acquired as a thin-slice protocol with metal-artefact reduction and, when component rotation is to be measured, including the hip and ankle as in standardised three-dimensional protocols [51], defines component position and rotation, subsidence, osteolysis and bone loss, and is particularly useful when component retention or compartmental addition is being considered. Magnetic resonance imaging (MRI) with metal-artefact-reduction sequences is indicated when the state of the cartilage, menisci or anterior cruciate ligament remains uncertain, and SPECT/CT may be added when the source of symptoms is unclear [51]. This determines the ceiling of anatomical preservation. Disease in two unresurfaced compartments closes every compartment-preserving pathway.

### 8.3. Step 3: Assess Bone Stock and Ligaments

Classify defects according to the Anderson Orthopaedic Research Institute (AORI) classification [43]. Type 1 defects generally permit primary components; Type 2 defects require stems and augments; Type 3 defects require revision constructs, cones or sleeves. Bone loss beyond Type 1 closes the compartment-preserving pathways. The thresholds used in this framework have limited reliability: inter-observer agreement for Ahlbäck grading of knee osteoarthritis was poor in one study (kappa approximately 0.1) [55], and we found no published inter-observer reliability data for the AORI classification in failed UKA, so the AORI type should be confirmed intraoperatively after component and cement removal. Knee flexion thresholds are clinical measurements and are subject to measurement error.

Assess the anterior cruciate ligament, the collaterals and the extensor mechanism. Anterior cruciate ligament function is assessed clinically (Lachman and pivot-shift tests) and, where doubtful, by MRI or by direct inspection at operation; collateral competence is assessed by clinical examination and stress radiographs. An intact, functional ACL is required by the EKS consensus for patellofemoral addition [30] and is generally regarded as a prerequisite for the other compartment-preserving strategies. The evidence for this assumption is indirect: in primary UKA, a meta-analysis of observational studies found similar revision rates in selected ACL-deficient and ACL-intact knees, mainly in patients older than 60 years without anteroposterior instability [56], whereas persistent ACL deficiency in the native knee is associated with greater joint laxity [57]. We therefore regard a functional ACL as a strong recommendation rather than an absolute requirement for Pathways A and B; ACL deficiency with symptomatic instability favours conversion to TKA. ACL status does not by itself determine the constraint of a conversion to TKA, which depends on the posterior cruciate and collateral ligaments (Section 6.4).

### 8.4. Step 4: Contextualise Within Patient and Centre Factors

Patient and centre factors do not override the anatomical findings of Steps 1 to 3; they determine which of the anatomically possible options is most appropriate for the individual patient.

- Younger, higher-demand patients: bone stock and cruciate function carry disproportionate long-term value given the elevated lifetime revision risk. The most conservative strategy consistent with the anatomical findings should be preferred, while recognising that the EKS consensus positions patellofemoral addition for older, comorbid patients [30].
- Activity level and expectations: high functional demands increase the value of preserved cruciate function and native kinematics, but also the mechanical demands on retained and added partial implants; the limited evidence supporting compartment-preserving options should be part of the discussion with the patient.
- Older patients with significant comorbidity: minimising surgical magnitude and rehabilitation burden favours compartmental addition where the anatomical prerequisites are met [30].
- Rehabilitation capacity: revision within the medial compartment or compartmental addition imposes a smaller surgical and rehabilitation burden, whereas conversion with augments, stems or increased constraint implies a longer recovery; where rehabilitation capacity is limited, the smaller operation may be preferred if, and only if, its anatomical prerequisites are met.
- Previous surgery: multiple previous procedures, previous infection, stiffness or arthrofibrosis, previous incisions and retained hardware argue against a further partial procedure and in favour of a single definitive reconstruction.
- Sex: in the German registry, male sex was associated with a higher risk of septic revision (HR 1.69, 95% CI 1.26 to 2.26) but a lower risk of aseptic revision (HR 0.75, 95% CI 0.66 to 0.84) after primary UKA [11]; sex-disaggregated outcomes of revision strategies after failed UKA are rarely reported and should be reported in future studies [45].
- Unexplained pain as the indication: defer revision until non-operative avenues are exhausted.
- Surgeon experience and centre volume: an annual volume of up to 25 UKAs is associated with an HR of 1.86 (95% CI 1.58 to 2.19) for aseptic revision of primary UKA relative to 51 to 200 UKAs per year [11], and hospitals performing 11 or fewer Oxford UKAs per year have lower implant survival [12]. Compartment-preserving revision demands more, not less, than standard conversion. It should be undertaken only by surgeons experienced in both partial and revision knee arthroplasty. Where that expertise is unavailable, referral to a centre with that expertise should be considered, while conversion to TKA remains an appropriate option.

### 8.5. Hierarchy of Strategies

The framework defines a hierarchy ordered by anatomical disruption and irreversibility: partial component revision → full revision within the medial compartment in highly selected cases → compartmental addition for isolated progression → conversion to TKA. Conversion remains the appropriate endpoint for the largest share of failures, but it should be reached by reasoned exclusion of the more conservative options rather than adopted by default.

## 9. Discussion

The central observation of this review is a mismatch. Failure after medial UKA arises from a heterogeneous set of mechanisms with distinct compartmental and structural consequences, yet the surgical response in contemporary practice is close to uniform: conversion to TKA accounted for 1743 of 1948 revisions (89.48%) in the Australian registry [14] and 485 of 620 (78.23%) in a regional registry [4]. Conversion to TKA is applied to problems that are frequently unicompartmental or bicompartmental in nature, and in doing so it permanently removes the bone stock and cruciate function that justified the index operation.

Conversion to TKA is not an incorrect treatment: it is the appropriate option whenever local revision or compartment-preserving surgery cannot fully correct the failure mechanism, and it remains the standard of care for most failures. Where it is indicated it performs well: ten-year aseptic survivorship of 95.6% in specialist practice is similar to that of primary TKA and higher than that of revision TKA [33], and registry [14,19] and single-centre comparative data [18] show conversion to be markedly more reliable than repeat unicompartmental revision. Our argument is narrower: a failure-mode-driven approach may benefit a definable subgroup of patients, although this has not yet been tested in direct, long-term prospective comparisons with conversion.

That subgroup is defined by biology rather than by mechanics. Progression in an unresurfaced compartment accounts for the largest share of late failures (56% beyond ten years [16] and 39.5% overall in one large single-centre series [17]), and it frequently occurs with the medial components still well fixed [24,31]. In this situation, removal of a functioning implant may not be structurally necessary, although no direct, long-term prospective study has yet shown that retaining it leads to better outcomes than conversion.

The difficulty is that the evidence for the alternative is thin, and this review has been deliberate in not disguising it. Pathway B1 rests on an expert consensus statement [30] and on indirect data from primary bicompartmental arthroplasty [35,36,41]. Pathway B2 rests on one dedicated series of 27 knees followed for a mean of four years [31], one multicentre comparative study of staged bicompartmental arthroplasty in general (27 versus 30 revision TKAs) [24], a descriptive multicentre series [34] and indirect data from simultaneous bi-unicompartmental arthroplasty [37]. Only the comparative study compares a compartment-preserving strategy with conversion; it is not specific to lateral UKA added to a medial UKA, and neither pathway has long-term survivorship data. The functional advantages demonstrated for bicompartmental constructs in the primary setting [35,36] are biomechanically plausible reasons to expect benefit, but they are not evidence of it in the revision setting.

The statistical power of the key comparative studies is limited. With a two-sided α of 0.05 and 80% power, the comparison of 27 staged bicompartmental arthroplasties with 30 revision TKAs [24] could detect only standardised differences in d ≥ 0.76, the matched cohort of 25 versus 50 patients [36] d ≥ 0.70 and the gait study of 16 versus 20 patients [35] d ≥ 0.97 (calculated for this review with noncentral t distributions). Small and moderate differences therefore cannot be excluded, and “comparable” outcomes in these studies must not be read as equivalence. For the same reason, effect sizes and their confidence intervals, rather than *p*-values alone, should guide the interpretation of these studies [58].

Several sources of bias run consistently in one direction and should temper interpretation. The favourable series are small [24,31,32], and the dedicated series of lateral UKA addition is single-centre and designer-associated [31]. Patients selected for compartmental addition have, by definition, already demonstrated a satisfactory result from partial arthroplasty over several years; this is a selection effect that cannot be adjusted for, and that plausibly inflates the apparent benefit. Patients treated with compartment-preserving procedures are therefore a carefully selected group, and their outcomes cannot be compared directly with those of conversion to TKA, which is performed in a broader population with more advanced or more complex failure. Conversely, the unfavourable comparative data on repeat unicompartmental revision are shaped by confounding by indication, since that strategy has been used preferentially for acute infection and bearing dislocation, conditions with an intrinsic tendency to recur [18].

Registry evidence carries its own limitations. Registries classify revision indications inconsistently, capture no patient-reported outcomes in most cases, and cannot distinguish a technically excellent conversion from a poor one [13,19,22]. Agreement in the direction of effect across registries does not by itself establish its magnitude or precision. Table 3 therefore summarises the available effect estimates with their confidence intervals where reported: the estimates for repeat unicompartmental revision versus conversion are consistently unfavourable and of broadly similar magnitude (hazard, risk or failure ratios of approximately 2.6 to 6), although several sources report no confidence intervals and none is adjusted for the indication for revision.

A final theme cuts across all three pathways: a substantial proportion of the problem may be iatrogenic and preventable. Malposition reduced five-year survival from 96.2% to 85.1%, an approximately four-fold higher five-year failure proportion (14.9% versus 3.8%) [27]; an annual volume of up to 25 UKAs was associated with an HR of 1.86 (95% CI 1.58 to 2.19) for aseptic revision relative to centres performing 51 to 200 UKAs per year [11]; the majority of revised Oxford knees showed no radiographic reason for revision [13]. In our view, improving primary technique, primary indication, and the threshold for revision is likely to reduce the size of the problem this review addresses more than any refinement of revision strategy.

## 10. Limitations

The most important methodological limitation of this review is that the structured database search was designed but not executed. Sources were therefore identified through targeted, purposive searches and reference screening, without independent duplicate selection. Searches were limited to PubMed/MEDLINE and Google Scholar, and only English-language publications were considered. Relevant studies may have been missed, the selection of sources may itself have introduced bias, and no claim of completeness can be made; the conclusions should therefore be read as an evidence-informed synthesis rather than as the result of a systematic review. Further limitations apply. The evidence supporting compartment-preserving strategies remains limited, with no randomised trials and sparse comparative data, and relies partly on indirect evidence from primary bicompartmental arthroplasty. Registry data are subject to confounding by indication and heterogeneous definitions of revision. Finally, the proposed decision framework is evidence-informed but has not been prospectively validated and should not be considered a standalone decision tool.

## 11. Future Research

Future research should prioritise multicentre, prospective studies that directly compare compartmental addition, revision within the medial compartment and conversion to TKA in comparable patients, and that report long-term implant survival, re-revision rates, patient-reported functional scores and patient satisfaction. A standardised dataset for failed UKA, within national arthroplasty registries or as a dedicated multicentre registry, should record patient sex, the failure mechanism, the extent of bone loss, ligament status, implant type and fixation, the revision procedure performed (distinguishing partial from full component revision and compartmental addition), and long-term outcomes including patient-reported measures, using consistent definitions. Dedicated studies are also needed to evaluate isolated component revision in UKA. Finally, the proposed decision framework requires prospective multicentre validation before it can be considered an evidence-based clinical tool.

## 12. Conclusions

Failed medial UKA is not a homogeneous problem, and its management should not be homogeneous either. A proposed four-step framework, which has not yet been validated, may help to structure the decision: characterise the failure mechanism and its extent; map the compartments; assess bone stock and ligament competence; and contextualise within patient and centre factors.

Within that framework a hierarchy of strategies is available. Revision confined to the medial compartment preserves the most anatomy but carries a re-revision risk several times that of conversion and should be restricted to isolated, fully correctable failures in selected patients. Retention of a well-fixed medial UKA with addition of a patellofemoral arthroplasty or a lateral UKA is biomechanically attractive and supported by encouraging but very limited evidence; it is a reasonable option in narrowly defined circumstances and is not standard treatment. Conversion to TKA remains the most reliable and best-evidenced option for the majority of failures, but should be approached as a formal revision procedure rather than as a primary arthroplasty performed late.

Personalized revision does not mean selecting the smallest possible operation. It means selecting the least invasive reconstruction that reliably addresses the complete failure mechanism. Retaining a component that is part of the problem risks early failure; removing a component that is functioning well sacrifices bone stock and may compromise kinematics. The partial arthroplasty philosophy that justifies the index procedure does not end when that procedure fails: replace what is diseased, preserve what still works, and defer the irreversible until it is genuinely necessary.

## Figures and Tables

**Table 1:** Comparison of the three treatment pathways after failed medial unicompartmental knee arthroplasty. Strength-of-evidence statements are qualitative author appraisals based on levels of evidence [20]. KOOS Jr., Knee injury and Osteoarthritis Outcome Score for Joint Replacement. ACL, anterior cruciate ligament; AORI, Anderson Orthopaedic Research Institute; CPR, cumulative percent revision; IKS, International Knee Society score; OA, osteoarthritis; OKS, Oxford Knee Score; PF, patellofemoral; PJI, periprosthetic joint infection; TKA, total knee arthroplasty; UKA, unicompartmental knee arthroplasty.

Parameter	A. Revision Within the Medial Compartment	B. Addition of Another Partial Implant	C. Conversion to TKA
Indications	Isolated, mechanically explicable, fully correctable failure: polyethylene wear; bearing dislocation without malposition; single-component loosening with the counterpart stable	B1: symptomatic PF progression, full-thickness loss of the lateral facet [30]. B2: symptomatic isolated lateral tibiofemoral progression [31]	Multicompartmental disease; bone loss beyond AORI Type 1; loosening, malposition or subsidence; ligament insufficiency; fracture; recurrent failure; chronic PJI
Prerequisites	Retained components confirmed well fixed and correctly positioned; competent ACL and collaterals; AORI Type 1 bone; other compartments preserved	Medial UKA well fixed, well positioned and asymptomatic; ACL intact; disease confined to one additional compartment; third compartment preserved; alignment acceptable or correctable; implants compatible; infection excluded	None beyond the capacity to manage the defect encountered
Contraindications	Adjacent-compartment OA; ACL insufficiency; AORI ≥ Type 2; unexplained pain; chronic infection	Any doubt about fixation, position, ligament competence or the third compartment; multicompartmental disease; stiffness or flexion contracture; infection	Active uncontrolled infection (requires staged reconstruction); unexplained pain without a structural target
Bone and ligament preservation	Highest; both cruciates and all collaterals retained	High; only the added compartment resurfaced; both cruciates retained	Obligatory sacrifice of the ACL; bone loss at explantation and cement removal
Technical difficulty	Moderate; depends on establishing the mechanism preoperatively	High; retained components constrain access, alignment reference and implant choice; component conflict must be avoided	Moderate to high; joint line and landmark loss; 26.4% required augmentation, 25.9% bone grafting and 8% revision implants [9]
Subsequent conversion	Remains available; bone stock largely preserved	Remains available; two bone-sparing implants to remove	Endpoint; further revision is revision TKA
Outcomes	2-yr survivorship free of re-revision 75% [18]; 3-yr CPR 28% to 30% [14]; but 8.33% (95% CI 1.75% to 22.47%) failure at mean 8.3 yr in a highly selected series with IKS 93.81/90.77 [32]	B1: no direct outcome data. B2: 27 knees, mean 4 yr, significant functional improvement, no failures or revisions (0/27; 95% CI upper bound 12.77%) [31]; staged bicompartmental arthroplasty vs. revision TKA (27 vs. 30): comparable KOOS Jr. improvement, higher Knee Society scores, one reoperation [24]	10-yr aseptic survivorship 95.6% in specialist practice [33]; OKS 30.02 vs. 37.16 for primary TKA (*p* < 0.01) and revision rate 1.97 vs. 0.48 per 100 component-years (*p* < 0.05) at registry level [19]
Re-revision risk	High: approximately six-fold that of conversion at 2 years [18]	Unknown: no series large enough or followed long enough to estimate	Moderate: higher than primary TKA at registry level [19]; similar to primary TKA and lower than revision TKA in one specialist centre [33], but not different from revision TKA in a community registry [10]
Strength of evidence (qualitative)	Level III registry and comparative evidence for the elevated risk; Level IV case series for favourable outcomes in selected patients	B1: Level V expert opinion and indirect evidence. B2: one Level IV case series (*n* = 27) [31], one Level III comparative study of staged bicompartmental arthroplasty (27 vs. 30) [24], a descriptive multicentre series [34] and indirect evidence	Level III registry and comparative evidence, and one meta-analysis of comparative studies [8]

**Table 2:** Characteristics, key results and limitations of the principal studies informing this review. Studies marked “indirect” concern primary or simultaneous arthroplasty and are not direct evidence for revision strategy after failed unicompartmental knee arthroplasty. AOANJRR, Australian Orthopaedic Association National Joint Replacement Registry; BKA, bicompartmental knee arthroplasty; CPR, cumulative percent revision; EKS, European Knee Society; EPRD, German Arthroplasty Registry; FJS-12, Forgotten Joint Score-12; HKA, hip–knee–ankle; IKS, International Knee Society score; KM, Kaplan–Meier; NJR, National Joint Registry; NZJR, New Zealand Joint Registry; OKS, Oxford Knee Score; PJI, periprosthetic joint infection; PROM, patient-reported outcome measure; RTS, return to sport; TKA, total knee arthroplasty; TWAAG, Trent and Wales Arthroplasty Audit Group; UKA, unicompartmental knee arthroplasty; U2U, unicompartmental-to-unicompartmental; WOMAC, Western Ontario and McMaster Universities Osteoarthritis Index. Levels of evidence are reported according to Wright et al. [20]; no formal risk-of-bias tool was applied, and the Limitations column lists design features only. KOOS Jr., Knee injury and Osteoarthritis Outcome Score for Joint Replacement; KSS, Knee Society Score; MINORS, Methodological Index for Non-Randomized Studies; PFA, patellofemoral arthroplasty; sBiKA, staged bicompartmental knee arthroplasty.

Study	Design	*n*	Setting	Follow-Up	Key Results	Limitations
Szymski et al. 2025 [11]	Registry (EPRD)	36,861 UKA	Primary	Max 7 yr	Aseptic loosening 32.5% of revisions (tibial 19.0%); 8.7% revised at 7 yr; volume ≤ 25/yr HR 1.86 (95% CI 1.58 to 2.19) and 26 to 50/yr HR 1.43 (95% CI 1.22 to 1.69) vs. 51 to 200/yr; uncemented HR 1.38 (95% CI 1.15 to 1.66)	Registry; no PROMs; 7-yr ceiling; Level III
Di Martino et al. 2021 [4]	Regional registry	6453 UKA vs. 54,012 TKA	Primary + revision	15 yr	Revision 18.2% vs. 6.2%; re-revision 16/35 after repeat UKA vs. 61/485 after conversion	Registry; no PROMs; single region
Hang et al. 2010 [14]	Registry (AOANJRR)	1948 revisions	Revision	3 yr CPR	Repeat UKA 28% (95% CI 22 to 36) and 30% (95% CI 22 to 40) CPR vs. 10% (95% CI 8.3 to 12) for conversion; infection excluded	Registry; 3-yr horizon; no PROMs
Pearse et al. 2010 [19]	Registry (NZJR)	4284 UKA, 236 revised	Revision	1999 to 2008	Conversion revision rate 4× primary TKA (1.97 vs. 0.48/100 component-yr); OKS 30.02 vs. 37.16	Registry; U2U group *n* = 31; OKS response bias
Restrepo et al. 2025 [18]	Comparative cohort	241 knees (24 repeat UKA, 217 conversion)	Revision	2 yr	Re-revision-free survival 96% vs. 75% (*p* < 0.0001); reoperation-free 92% vs. 69%; re-revision causes PJI 57%, dislocation 28%	Single centre; repeat-UKA arm *n* = 24; confounding by indication (71% acute PJI)
Epinette et al. 2014 [32]	Multicentre case–control	36 of 425 revised	Revision (UKA→UKA)	Mean 8.3 ± 6.7 yr	3 (8.33%; 95% CI 1.75% to 22.47%) iterative revision at 8 mo, 9 mo, 3 yr; IKS 93.81 ± 9.26/90.77 ± 15.41	Small; highly selected; authors state KM survival analysis not possible
Pandit et al. 2017 [31]	Consecutive series	27 knees/25 patients	B2: lateral UKA added after medial UKA	Mean 4 ± 1.9 yr; 8.1 ± 4.6 yr between procedures	Significant functional improvement (paired *t*-test, *p* = 0.003; effect size not reported); no radiological failure, no reoperation (0/27; 95% CI upper bound 12.77%); median stay 3 days	*n* = 27; single designer-associated centre; no comparator; no survivorship analysis; Level IV
Haffar et al. 2022 [24]	Retrospective multicentre comparative	27 sBiKA/30 revision TKA	Staged addition of a second partial arthroplasty vs. conversion	Mean 5.7 ± 3 vs. 3.2 ± 2 yr	KOOS Jr. improved by an equivalent amount; KSS objective 90.4 ± 10 vs. 72.1 ± 20 and function 80.3 ± 18 vs. 67.1 ± 19; one reoperation after sBiKA	Non-consecutive; unequal follow-up; detectable d ≥ 0.76; not specific to lateral UKA after medial UKA; Level III
Jones et al. 2025 [30]	Delphi consensus (EKS)	49 → 42 members	B1: PFA added to UKA	n/a	83% agreement on six indications incl. lateral-facet full-thickness loss, intact ACL, Ahlbäck ≤ 1 laterally, flexion ≥ 100°, older comorbid patients	Expert opinion, not outcome evidence; single society; not externally validated
Garner et al. 2023 [35]	Cross-sectional gait study	16 BKA/20 TKA/19 healthy	Primary arthroplasty (indirect)	Cross-sectional	Top walking speed 24% faster than TKA (*p* < 0.001); OKS 41 vs. 38 (*p* < 0.02)	*n* = 16; primary not revision; single centre; Level III
Deng et al. 2023 [36]	Matched cohort 1:2	25 BKA/50 TKA	Primary arthroplasty (indirect)	Min 2 yr	WOMAC-function 97.1 vs. 89.7 (*p* < 0.001); FJS-12 89.6 vs. 53.1 (*p* < 0.001); RTS 71.6% vs. 56.5% (*p* = 0.039)	Small; retrospective; primary not revision
Akkawi et al. 2023 [37]	Systematic review	7 studies, 302 surgeries	Simultaneous bi-UKA (indirect)	Variable	Survival 83% to 100%; 22 revisions, 16 for aseptic loosening; 8 intraoperative fractures	Simultaneous primary, not staged addition; small heterogeneous studies
Nwankwo et al. 2026 [33]	Database cohort	439 conversions	Revision	Mean 5.7 yr	10-yr aseptic survivorship 95.6% vs. 95.8% primary vs. 84.2% revision TKA; failure mode not influential (*p* = 0.80)	Single high-volume centre; selected specialist-practice cohort; external validity uncertain
Khan et al. 2013 [9]	Registry audit (TWAAG)	201 conversions	Revision	n/a (audit)	Augmentation 26.4%; grafting 25.9%; 78% cruciate-retaining primary implants	No clinical or functional outcomes; older implant era
Aleto et al. 2008 [7]	Consecutive series	32 revisions	Revision	n/a	Medial tibial collapse 47%; 87% all-polyethylene; 7/15 failed <16 months	Small; single centre; historical all-poly implants
Kennedy et al. 2020 [13]	Blinded radiographic review (NJR)	107 revised Oxford UKA	Revision	n/a	No reason for revision seen in 67%	Radiographic only; no clinical or aspiration data
Innocenti et al. 2025 [38]	Systematic review + meta-analysis	20 studies, 795 cases	Revision	To 31 Oct 2024	revTKA: HKA outliers 13.3% vs. 26.1%. revUKA: outcomes generally similar, mixed alignment results	Level IV; MINORS-rated; heterogeneous

**Table 3:** Effect estimates for re-revision after revision of a failed UKA, as reported in the sources or calculated for this review (*). Values are percentages, ratios or rates with 95% confidence intervals where available. CPR, cumulative percent revision (excluding revisions for infection in [14]); HR, hazard ratio adjusted for age and sex; RR, crude risk ratio; NZJR, New Zealand Joint Registry; AOANJRR, Australian Orthopaedic Association National Joint Replacement Registry.

Source	Comparison	End Point	Estimate (95% CI)	Comment
AOANJRR [14]	Major UKA-to-UKA vs. conversion to TKA	3-yr CPR; HR	28 (22 to 36) vs. 10 (8.3 to 12); HR 2.6 (1.9 to 3.6)	Registry; adjusted for age and sex
AOANJRR [14]	Minor UKA-to-UKA vs. conversion to TKA	3-yr CPR; HR	30 (22 to 40) vs. 10 (8.3 to 12); HR 4.8 (3.1 to 7.5) at 0 to 1.5 yr, 1.8 (0.95 to 3.6) thereafter	Time-varying hazard
AOANJRR [14]	Revision TKA (both components) vs. conversion to TKA	3-yr CPR; HR	12 (9.0 to 15) vs. 10 (8.3 to 12); HR 1.3 (0.95 to 1.6)	No statistically significant difference; the CI does not exclude a higher risk after revision TKA
NZJR [19]	Conversion to TKA vs. primary TKA	Revisions per 100 component-years	1.97 vs. 0.48 (ratio approximately 4); CI not reported	205 conversions
NZJR [19]	UKA-to-UKA vs. primary TKA	Rate ratio	Approximately 13; rate and CI not reported	31 knees
Regional registry [4]	Repeat UKA vs. conversion to TKA	Proportion re-revised	16/35 (45.71%) vs. 61/485 (12.58%); RR 3.63 (2.36 to 5.59) *	Crude; not adjusted for follow-up time
Single centre [18]	UKA-to-UKA vs. conversion to TKA	2-yr survival free of re-revision	75% vs. 96%; CI not reported	71% of UKA-to-UKA revisions for acute PJI
Three centres [23]	Conversion to TKA	Re-revision	9/175 (5.14%; 2.38 to 9.54) *; 1.23 per 100 observed component-years	Reported as 4.5% in the source
Specialist centre [33]	Conversion vs. primary TKA	10-yr aseptic survivorship	95.6% (91.7 to 97.7) vs. 95.8% (95.3 to 96.3)	Single high-volume centre; revision TKA 84.2%

## Data Availability

No new data were created in this study. All data discussed are available in the cited publications.

## Supplementary Materials

The following supporting information can be downloaded at: https://www.mdpi.com/article/10.3390/jcm15218577/s1, File S1: Database-specific structured search strategies (designed but not executed).

## Abbreviations

ACL	anterior cruciate ligament
AI	artificial intelligence
AOANJRR	Australian Orthopaedic Association National Joint Replacement Registry
AORI	Anderson Orthopaedic Research Institute
BKA	bicompartmental knee arthroplasty
CI	confidence interval
CPR	cumulative percent revision
CRP	C-reactive protein
CT	computed tomography
DAIR	debridement, antibiotics and implant retention
EBJIS	European Bone and Joint Infection Society
EKS	European Knee Society
EPRD	German Arthroplasty Registry (Endoprothesenregister Deutschland)
ESR	erythrocyte sedimentation rate
FJS-12	Forgotten Joint Score-12
GRADE	Grading of Recommendations Assessment, Development and Evaluation
HKA	hip–knee–ankle
HR	hazard ratio
IKS	International Knee Society score
KM	Kaplan–Meier
KOOS Jr.	Knee injury and Osteoarthritis Outcome Score for Joint Replacement
KSS	Knee Society Score
MINORS	Methodological Index for Non-Randomized Studies
MRI	magnetic resonance imaging
NJR	National Joint Registry
NZJR	New Zealand Joint Registry
OA	osteoarthritis
OKS	Oxford Knee Score
PF	patellofemoral
PFA	patellofemoral arthroplasty
PJI	periprosthetic joint infection
PMN	polymorphonuclear
PRISMA	Preferred Reporting Items for Systematic Reviews and Meta-Analyses
PROM	patient-reported outcome measure
RR	risk ratio
RTS	return to sport
SANRA	Scale for the Assessment of Narrative Review Articles
sBiKA	staged bicompartmental knee arthroplasty
SPECT/CT	single-photon emission computed tomography combined with computed tomography
TKA	total knee arthroplasty
TWAAG	Trent and Wales Arthroplasty Audit Group
U2U	unicompartmental-to-unicompartmental
UKA	unicompartmental knee arthroplasty
WCC	white cell count
WOMAC	Western Ontario and McMaster Universities Osteoarthritis Index
