# Supplementary material for: Personalized Revision Arthroplasty After Failed Medial Unicompartmental Knee Arthroplasty: A Contemporary Narrative Review
Source: J Clin Med. 2026 Nov 3;15(21):8577. doi: 10.3390/jcm15218577 (PMC13763757; doi:10.3390/jcm15218577)
Supplement: Supplementary file 1 [file jcm-15-08577-s001.zip › jcm-4564627-supplementary.pdf]

Supplementary File S1. Database-specific structured search strategies (designed but not executed)

Personalized Revision Arthroplasty After Failed Medial Unicompartmental Knee Arthroplasty: A Contemporary Narrative Review

The search strategies below were designed to define the scope of the review explicitly and to allow the search to be reproduced or updated in a future systematic review. They were not executed as a formal systematic search: no database-level record counts, deduplication or independent duplicate screening were performed, and no study-flow diagram is therefore presented (Section 2.2 of the manuscript).

The sources actually used in the review were identified through targeted searches of PubMed/MEDLINE and Google Scholar and by screening the reference lists of relevant reviews and registry reports, as described in Section 2.3 of the manuscript.

Concept blocks

Block	Concept	Role
**A**	Medial unicompartmental, unicondylar or partial knee arthroplasty	Population (index procedure)
**B**	Failure, revision, conversion, reoperation, disease progression and named failure mechanisms	Problem
**C**	Treatment destinations: conversion to TKA, repeat medial UKA, component or bearing exchange, addition of patellofemoral arthroplasty, addition of lateral UKA, bi-unicompartmental reconstruction	Intervention or comparator

- Block A is restricted to medial UKA (primary run). Because many studies describe a medial cohort only as "UKA", each strategy also contains an unrestricted population block for a sensitivity run; records retrieved only by the sensitivity run would be eligible only if the full text confirmed a medial index procedure.
- Block C contains explicit terms for the addition of a lateral UKA, which is the only way to retrieve the sparse literature on Pathway B2; these terms also retrieve primary lateral UKA series, which are outside the scope of the review.
- Intended limits: English language; humans; no date restriction (coverage from database inception to 15 August 2026). Conference abstracts were to be excluded.

S1.1. PubMed/MEDLINE (pubmed.ncbi.nlm.nih.gov)

```
#A  ("medial unicompartmental knee arthroplasty"[tiab]
     OR "medial unicompartmental knee replacement"[tiab]
     OR "medial unicondylar knee arthroplasty"[tiab]
     OR "medial unicondylar knee replacement"[tiab]
     OR "medial partial knee arthroplasty"[tiab]
     OR "medial partial knee replacement"[tiab]
     OR "medial UKA"[tiab] OR "medial UKR"[tiab]
     OR ("Oxford"[tiab] AND ("unicompartmental"[tiab] OR "unicondylar"[tiab])))

// unrestricted population block (sensitivity run)
#A2 ("Arthroplasty, Replacement, Knee"[Mesh]
     OR "unicompartmental knee arthroplasty"[tiab]
     OR "unicompartmental knee replacement"[tiab]
     OR "unicondylar knee arthroplasty"[tiab]
     OR "unicondylar knee replacement"[tiab]
     OR "partial knee arthroplasty"[tiab]
     OR "partial knee replacement"[tiab]
```

```
     OR UKA[tiab] OR UKR[tiab] OR PKA[tiab] OR PKR[tiab])

#B  (failure[tiab] OR failed[tiab] OR failing[tiab]
     OR revision*[tiab] OR revised[tiab] OR re-revision*[tiab] OR rerevision*[tiab]
     OR conversion[tiab] OR converted[tiab]
     OR reoperation*[tiab] OR "re-operation"[tiab]
     OR survivorship[tiab] OR "implant survival"[tiab]
     OR loosening[tiab] OR osteolysis[tiab] OR subsidence[tiab] OR migration[tiab]
     OR "bearing dislocation"[tiab] OR "bearing exchange"[tiab]
     OR "polyethylene wear"[tiab] OR "insert exchange"[tiab] OR "liner exchange"[tiab]
     OR malposition[tiab] OR malalignment[tiab] OR instability[tiab]
     OR "unexplained pain"[tiab] OR "persistent pain"[tiab]
     OR "periprosthetic joint infection"[tiab] OR "prosthetic joint infection"[tiab]
     OR PJI[tiab]
     OR "periprosthetic fracture"[tiab] OR "tibial fracture"[tiab]
     OR "tibial collapse"[tiab]
     OR "osteoarthritis progression"[tiab] OR "progression of osteoarthritis"[tiab]
     OR "adjacent compartment"[tiab] OR "lateral compartment"[tiab]
     OR "patellofemoral"[tiab])

#C  ("total knee arthroplasty"[tiab] OR "total knee replacement"[tiab] OR TKA[tiab]
    OR TKR[tiab]
     OR "revision total knee"[tiab]
     OR "component exchange"[tiab] OR "component revision"[tiab]
     OR "single component"[tiab] OR "isolated tibial"[tiab] OR "isolated femoral"[tiab]
     OR bicompartmental[tiab] OR "bi-compartmental"[tiab]
     OR "bi-unicompartmental"[tiab] OR "biunicompartmental"[tiab]
     OR "bi-unicondylar"[tiab]
     OR "lateral unicompartmental knee arthroplasty"[tiab]
     OR "lateral unicompartmental knee replacement"[tiab]
     OR "lateral compartment arthroplasty"[tiab]
     OR "lateral UKA"[tiab] OR "lateral UKR"[tiab]
     OR "second compartment"[tiab] OR "added compartment"[tiab]
     OR "staged bicompartmental"[tiab] OR "sequential unicompartmental"[tiab]
     OR "patellofemoral arthroplasty"[tiab] OR "patellofemoral replacement"[tiab]
     OR "trochlear"[tiab]
     OR augment*[tiab] OR stem*[tiab] OR cone*[tiab] OR sleeve*[tiab]
     OR "bone graft"[tiab])

#D  #A AND #B AND #C   // primary run (medial-restricted)
#E  #D AND (english[Filter]) AND (humans[Filter])

#F  #A2 AND #B AND #C   // sensitivity run (unrestricted population)
#G  #F AND (english[Filter]) AND (humans[Filter])
#H  #G NOT #E   // records retrieved only by the sensitivity run
```

S1.2. Embase (Elsevier, embase.com)

```
#1  'unicompartmental knee arthroplasty'/exp
#2  'knee hemiarthroplasty'/exp
#3  ('medial unicompartmental knee arthroplast*'
    OR 'medial unicompartmental knee replacement*'
     OR 'medial unicondylar knee arthroplast*' OR 'medial partial knee arthroplast*'
     OR 'medial UKA' OR 'medial UKR'
     OR (Oxford AND (unicompartmental OR unicondylar))):ti,ab,kw
#3b ('unicompartmental knee arthroplast*' OR 'unicompartmental knee replacement*'
     OR 'unicondylar knee arthroplast*' OR 'unicondylar knee replacement*'
     OR 'partial knee arthroplast*' OR 'partial knee replacement*'
     OR UKA OR UKR OR PKA OR PKR):ti,ab,kw
#4  #3   // medial-restricted population block (primary run)
#4b #1 OR #2 OR #3b   // unrestricted population block (sensitivity run)

#5  ('prosthesis failure'/exp OR 'reoperation'/exp OR 'prosthesis loosening'/exp)
#6  (failure OR failed OR revision* OR revised OR 're-revision*' OR conversion
    OR converted
     OR reoperation* OR survivorship OR loosening OR osteolysis OR subsidence OR migration
     OR 'bearing dislocation' OR 'bearing exchange' OR 'polyethylene wear'
     OR 'insert exchange' OR 'liner exchange' OR malposition OR malalignment
     OR instability
     OR 'unexplained pain' OR 'periprosthetic joint infection'
     OR 'periprosthetic fracture'
     OR 'tibial collapse' OR 'osteoarthritis progression'
     OR 'adjacent compartment'):ti,ab,kw
#7  #5 OR #6

#8  ('total knee arthroplasty'/exp OR 'knee prosthesis'/exp)
#9  ('total knee arthroplast*' OR 'total knee replacement*' OR TKA OR TKR
```

```
     OR 'component exchange' OR 'component revision' OR 'single component'
     OR bicompartmental OR 'bi-compartmental' OR 'bi-unicompartmental' OR 'bi-unicondylar'
     OR 'lateral unicompartmental knee arthroplast*'
     OR 'lateral unicompartmental knee replacement*'
     OR 'lateral compartment arthroplast*' OR 'lateral UKA' OR 'lateral UKR'
     OR 'staged bicompartmental' OR 'sequential unicompartmental' OR 'second compartment'
     OR 'patellofemoral arthroplast*' OR 'patellofemoral replacement*' OR trochlear
     OR augment* OR stem* OR cone* OR sleeve* OR 'bone graft*'):ti,ab,kw
#10 #8 OR #9

#11  #4  AND #7 AND #10   // primary run (medial-restricted)
#11b #4b AND #7 AND #10   // sensitivity run (unrestricted population)
#12 #11 AND [english]/lim AND [humans]/lim
#13 #12 NOT ('conference abstract'/it OR 'conference review'/it OR 'editorial'/it
    OR 'note'/it)
#14 (#11b AND [english]/lim AND [humans]/lim) NOT #12
```

S1.3. Scopus (Elsevier, scopus.com)

```
( TITLE-ABS-KEY ( "medial unicompartmental knee arthroplast*"
    OR "medial unicompartmental knee replacement*"
    OR "medial unicondylar knee arthroplast*" OR "medial partial knee arthroplast*"
    OR "medial UKA" OR "medial UKR"
    OR ( Oxford AND ( unicompartmental OR unicondylar ) ) ) )
AND
( TITLE-ABS-KEY ( failure OR failed OR revision* OR revised OR conversion OR converted
    OR reoperation* OR survivorship OR loosening OR osteolysis OR subsidence
    OR "bearing dislocation" OR "polyethylene wear" OR "insert exchange"
    OR malposition OR malalignment OR instability OR "unexplained pain"
    OR "periprosthetic joint infection" OR "periprosthetic fracture" OR "tibial collapse"
    OR "osteoarthritis progression" OR "adjacent compartment" ) )
AND
( TITLE-ABS-KEY ( "total knee arthroplast*" OR "total knee replacement*" OR TKA OR TKR
    OR "component exchange" OR "component revision" OR bicompartmental
    OR "bi-unicompartmental" OR "bi-unicondylar"
    OR "lateral unicompartmental knee arthroplast*" OR "lateral compartment arthroplast*"
    OR "lateral UKA" OR "lateral UKR"
    OR "staged bicompartmental" OR "sequential unicompartmental" OR "second compartment"
    OR "patellofemoral arthroplast*"
    OR trochlear OR augment* OR stem* OR cone* OR sleeve* OR "bone graft*" ) )
AND ( LIMIT-TO ( LANGUAGE , "English" ) )
AND ( LIMIT-TO ( DOCTYPE , "ar" ) OR LIMIT-TO ( DOCTYPE , "re" ) )
AND ( LIMIT-TO ( SUBJAREA , "MEDI" ) )
```

Sensitivity version: the first block is replaced by the unrestricted population terms ("unicompartmental knee arthroplast" OR "unicompartmental knee replacement*" OR "unicondylar knee arthroplast*" OR "partial knee arthroplast*" OR UKA OR UKR).*

S1.4. Cochrane Library (Wiley; CDSR and CENTRAL)

```
#1  MeSH descriptor: [Arthroplasty, Replacement, Knee] explode all trees
#2  ("medial unicompartmental knee arthroplast*" OR "medial unicondylar knee arthroplast*"
     OR "medial partial knee arthroplast*" OR "medial UKA" OR "medial UKR"):ti,ab,kw
#2b ("unicompartmental knee arthroplast*" OR "unicompartmental knee replacement*"
     OR "unicondylar knee arthroplast*" OR "partial knee arthroplast*"
     OR "partial knee replacement*" OR UKA OR UKR):ti,ab,kw
#3  #2   // medial-restricted population block (primary run)
#3b #1 OR #2b   // unrestricted population block (sensitivity run)
#4  (failure OR failed OR revision* OR conversion OR reoperation* OR loosening
     OR "bearing dislocation" OR "polyethylene wear" OR "insert exchange"
     OR malposition OR instability OR "unexplained pain"
     OR "periprosthetic joint infection" OR "periprosthetic fracture"
     OR "osteoarthritis progression"):ti,ab,kw
#5  ("total knee arthroplast*" OR "total knee replacement*" OR TKA OR TKR
     OR bicompartmental OR "bi-unicompartmental" OR "patellofemoral arthroplast*"
     OR "lateral unicompartmental knee arthroplast*" OR "lateral compartment arthroplast*"
     OR "staged bicompartmental" OR "sequential unicompartmental"
     OR augment* OR stem* OR cone* OR sleeve*):ti,ab,kw
#6  #3  AND #4 AND #5   // primary run
#6b #3b AND #4 AND #5   // sensitivity run
```
